# Staphylococci planktonic and biofilm environments differentially affect osteoclast formation

**DOI:** 10.1007/s00011-023-01745-9

**Published:** 2023-06-17

**Authors:** Elisabeth Seebach, Franziska V. Kraus, Tabea Elschner, Katharina F. Kubatzky

**Affiliations:** 1 Department of Infectious Diseases, Medical Microbiology and Hygiene, Heidelberg University, Im Neuenheimer Feld 324, 69120 Heidelberg, Germany; 2 Department of Internal Medicine 5 - Hematology Oncology Rheumatology, Heidelberg University Hospital, Im Neuenheimer Feld 410, 69120 Heidelberg, Germany; 3 Present Address: Institute for Cardiovascular Sciences and Institute of Neurovascular Cell Biology (INVZ), University Hospital Bonn, University of Bonn, Bonn, Germany

**Keywords:** Implant-related bone infections, Staphylococcus, Biofilm, Macrophages, Immune response, Osteoclastogenesis

## Abstract

**Introduction:**

The pathophysiology of chronic implant-related bone infections is characterized by an increase in osteoclast numbers and enhanced bone resorption. Biofilms are a major reason for chronicity of such infections as the biofilm matrix protects bacteria against antibiotics and impairs the function of immune cells. Macrophages are osteoclast precursor cells and therefore linked to inflammation and bone destruction.

**Objective and method:**

Investigations on the impact of biofilms on the ability of macrophages to form osteoclasts are yet missing and we, therefore, analyzed the effect of *Staphylococcus aureus* (SA) and *Staphylococcus epidermidis* (SE) planktonic and biofilm environments on osteoclastogenesis using RAW 264.7 cells and conditioned media (CM).

**Results:**

Priming with the osteoclastogenic cytokine RANKL before CM addition enabled the cells to differentiate into osteoclasts. This effect was highest in SE planktonic or SA biofilm CM. Simultaneous stimulation with CM and RANKL, however, suppressed osteoclast formation and resulted in formation of inflammation-associated multinucleated giant cells (MGCs) which was most pronounced in SE planktonic CM.

**Conclusion:**

Our data indicate that the biofilm environment and its high lactate levels are not actively promoting osteoclastogenesis. Hence, the inflammatory immune response against planktonic bacterial factors through Toll-like receptors seems to be the central cause for the pathological osteoclast formation. Therefore, immune stimulation or approaches that aim at biofilm disruption need to consider that this might result in enhanced inflammation-mediated bone destruction.

**Supplementary Information:**

The online version contains supplementary material available at 10.1007/s00011-023-01745-9.

## Introduction

Chronic implant-related bone infections are associated with tissue inflammation and bone destruction through bone resorbing osteoclasts. This ultimately leads to implant loosening and loss of implant function [1, 2]. Staphylococci, primarily *Staphylococcus aureus* (SA) and *Staphylococcus epidermidis* (SE), belong to the most frequently isolated bacteria. They can colonize the implant and form biofilms on its surface. Within the biofilm matrix, bacteria are protected against most antibiotics as well as the host immune response [3–6]. Furthermore, the biofilm metabolic environment is associated with a rather anti-inflammatory and tolerogenic immune polarization [7, 8]. Thus, these bacteria frequently cause chronic infections, which often results in the removal of the implant as the only possible treatment option [9, 10].

Macrophages are innate immune cells and possess a dual role in bone infections. They belong to the initial host defense against invading pathogens, but also serve as precursor cells for bone resorbing osteoclasts [11]. Osteoclastogenesis is induced by binding of receptor activator of NF-κB ligand (RANKL) to its receptor RANK on the osteoclast precursor cell surface. Downregulation of the transcription factor interferon regulatory factor 8 (IRF8) then allows the activation and auto-amplification of the osteoclast master regulator nuclear factor of activated T cells 1 (NFATc1) [12]. Ultimately, activation of NFATc1 signaling causes the subsequent induction of osteoclastogenic genes associated with cell fusion and bone resorption activity [13, 14]. Pro-inflammatory environments can promote osteoclastogenic differentiation of macrophages due to an inflammation-induced increase of the production of RANKL by immune cells, fibroblasts, and osteoblasts [15, 16]. In the case of bone infections, it is known that the release of pro-inflammatory cytokines such as tumor necrosis factor (TNF)-α, interleukin (IL)-1β and IL-6 by immune cells and the subsequent production of RANKL by osteoblasts promotes osteoclast formation and activity [17, 18]. The release of pro-inflammatory cytokines is mediated as a consequence of pattern recognition receptor (PRR) activation by bacterial factors. In case of implant-related bone infections which are mainly caused by staphylococci, activation of Toll-like receptors TLR-2 and TLR-9 plays an important role [19]. Additionally, the contribution of other bacterial factors such as Protein A of SA was described to promote osteoclast formation [20, 21]. The effect of TLR stimulation seems to depend on the differentiation state of the precursor cell. While this is inhibitory for RANKL-induced osteoclastogenesis of uncommitted macrophages which have not been activated by RANKL or TLR ligands, it is stimulatory for osteoclast maturation of RANKL-primed osteoclast progenitors [22]. Planktonic infections are associated with a TLR-mediated pro-inflammatory immune response that is able to initiate bacterial clearance. Biofilm infections, however, lead to reduced TLR activation and an insufficient immune response [4]. The different extent in immune activity between planktonic and biofilm conditions might influence the fate decision of macrophages toward immune effector cell versus osteoclast. In addition, also the metabolism of biofilm bacteria is discussed to skew the local environment toward glucose deprivation and lactate enrichment by influencing host cell metabolism and cellular function [23]. Osteoclast differentiation relies on increased glycolysis and glycolysis-derived lactate is involved in the resorptive activity of osteoclasts [24]. Therefore, a direct effect of the biofilm metabolic environment on osteoclast formation is possible.

The purpose of our study was to determine if changes of the bacterial environment during biofilm formation affect the fate decision of macrophages toward immune effector cell activity or osteoclast differentiation. We included SA and SE as the two most relevant bacteria causing implant-related bone infections [6, 9]. Both share the ability to form biofilms but differ regarding their virulence. SA uses a broad range of pathogenicity factors, is highly virulent, and therefore is associated with acute infections [25]. The commensal SE mainly relies on biofilm formation as immune evasion strategy and induces low-grade inflammation and chronic infection [26]. To clarify the effect of a biofilm environment on macrophage immune activation and osteoclastogenesis, we treated murine RAW 264.7 macrophages with conditioned media (CM) generated from SA or SE planktonic and biofilm cultures in the presence of RANKL. We evaluated the immune activation of the cells as well as their ability to form osteoclasts. Furthermore, we investigated potential mechanisms that could mediate the CM effects on macrophage differentiation.

## Materials and methods

### Bacteria culture and preparation of conditioned media

*Staphylococcus aureus* strain ATCC 49230 (UAMS-1, isolated from a patient with chronic osteomyelitis) [27] and *Staphylococcus epidermidis* strain DSM 28319 (RP62A, isolated from a catheter sepsis) were used for preparation of conditioned media which was done as previously described [28]. Both strains were successfully used in implant-related bone infection models and resulted in the expected clinical symptoms of acute (SA) and low-grade (SE) inflammation, respectively [29, 30]. Furthermore, both strains can form biofilms in vitro [31, 32], and therefore are suitable to investigate the effects of the bacterial environments on macrophage function in the context of a bone infection. Bacteria were cultivated on Columbia agar plates with 5% sheep blood (BD, Germany). Three to five colonies were transferred into trypticase soy bouillon (TSB; BD, Germany) and cultivated under shaking for 3 h at 37 °C to receive log-phase bacteria which are in exponential growth. Bacteria concentration was adjusted to 6*10^5^ CFU/ml in Dulbecco's modified Eagle medium (DMEM) high glucose (Anprotec, Germany) + 10% heat-inactivated fetal calf serum (FCS; Biochrom GmbH, Germany). For planktonic cultures, bacteria were cultivated under shaking (200 rpm) for 24 h at 37 °C and 5% CO_2_. For biofilm cultures, bacteria were plated in 24 well with 1 ml per well and cultivated under static conditions for 3 or 6 days with medium replacement every 24 h. Medium was harvested after 24 h of planktonic culture or the last 24 h medium change before day 3 or 6 biofilm culture by centrifugation at 4000 rpm for 15 min at 4 °C. Supernatants were sterile filtered through a 0.2 µm filter. pH of CM was adjusted to physiological pH of growth medium (DMEM high glucose + 10% FCS). Aliquots were stored at − 80 °C. Planktonic and biofilm CM from the same approach were compared within one experiment. For the unstimulated CM control, the growth medium (DMEM high glucose + 10% FCS) of the respective approach was treated similar to CM but without bacteria inoculation. In vitro biofilm formation capacity of SA and SE strains and the characteristics of the CM were evaluated in a previous study showing that the generated CM represent the in vivo biofilm environment [28].

### Free bacterial DNA content in CM

DNA was extracted from 1 ml of three representative CM approaches, respectively, using the DNeasy^®^ Blood and Tissue Kit from Qiagen, Germany. Without prior cell lysis, 500 µl of 100% ethanol were directly added to the sample. The following steps were done according to the manufacturer’s protocol. DNA was eluted in 30 µl elution buffer and 12 µl of the DNA eluate was used for qPCR. DNA was amplified for 40 cycles using SA-specific primers for gyrase B (*gyrB*; SAUSA300_0005, fw: AGTAACGGATAACGGACGTGGTA, rev: CCAACACCATGTAAACCACCAGAT) and SE-specific primers for gyrase A (*gyrA*; SERP2548, fw: AGCAGCAGGTGTGAAAGGTA, rev: TACGCTCGGTAATTGTCGCA). SA-specific primers for *gyrB* were designed based on the genome sequence of *Staphylococcus aureus subsp. aureus USA300_FPR3757* (GCA_000013465). SE-specific primers for *gyrA* were designed based on the genome sequence of *Staphylococcus epidermidis RP62A* (GCA_000011925). Genome sequences were extracted from the Ensembl Bacteria genome browser and primers were obtained from biomers.net GmbH, Germany. PCR products were visualized by agarose gel electrophoresis.

### Cell culture and stimulation of macrophages

**Fig. 1:**
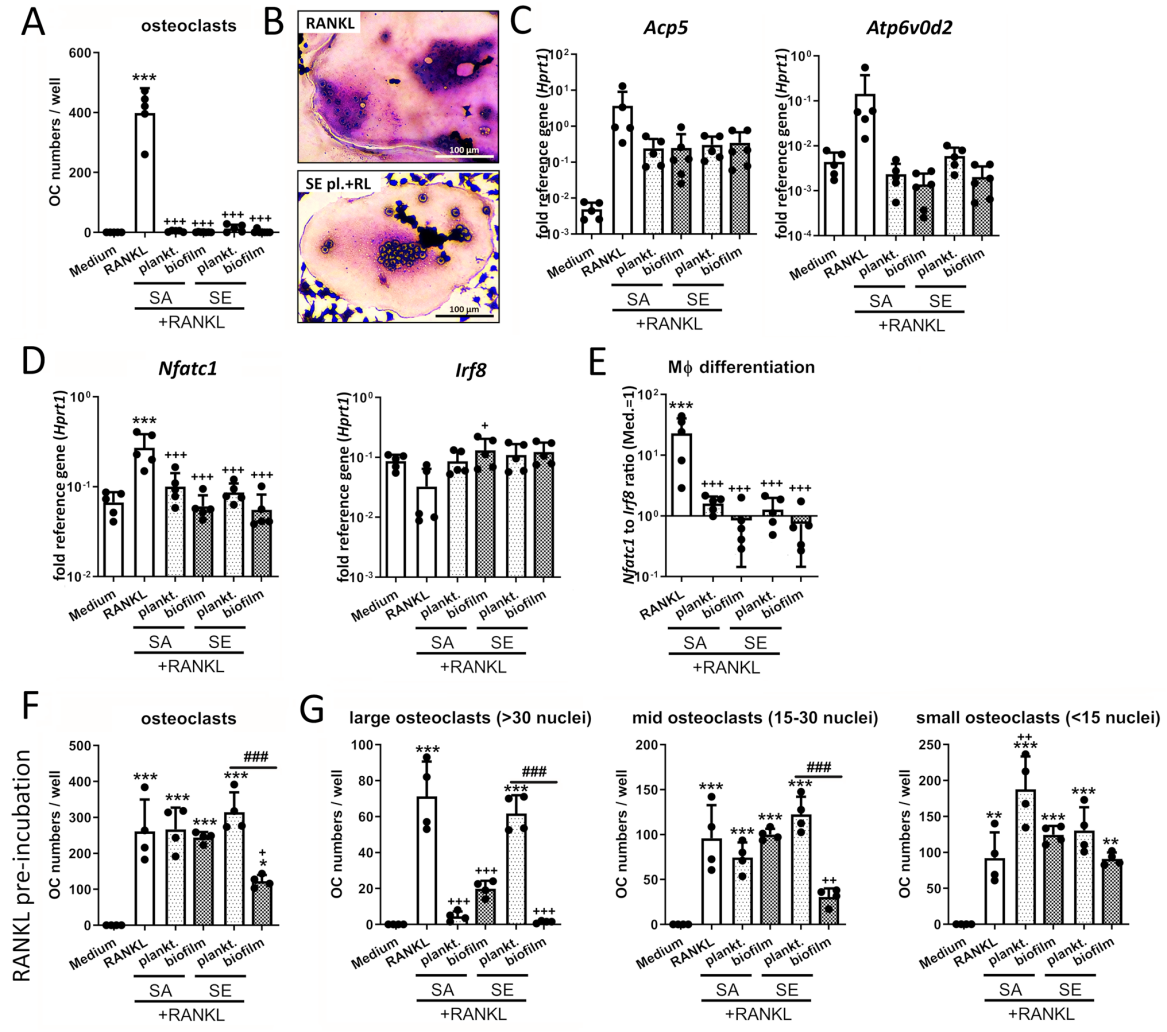
Effect of CM on RANKL-mediated osteoclastogenesis of macrophages. RAW 264.7 cells were cultivated in CM 1:1 diluted in fresh growth media (DMEM high glucose + 10% FCS + 1% Pen/Strep) and RANKL-induced osteoclastogenic differentiation was investigated. **A** Osteoclast formation of unprimed macrophages. Cells were stimulated with CM + RANKL (50 ng/ml) for 5 days. On day 5, cells were fixed, stained for TRAP and nuclei, and total numbers of formed osteoclasts (OCs) per well were counted. Osteoclasts were defined as TRAP-positive multinucleated giant cells with at least three nuclei. Data are presented as OC numbers per well. *n* = 5 experiments in duplicates (mean of duplicates was included in statistics). **B** Visualization of formed osteoclasts after 5 days of differentiation. TRAP activity is shown by violet color development and nuclei are stained in blue. Upper picture shows RANKL control, lower picture shows one of the few osteoclasts formed after treatment with RANKL + SE planktonic CM. **C** Gene expression analysis of osteoclast marker genes *Acp5* and *Atp6v0d2*. Cells were stimulated with CM + RANKL (50 ng/ml) for 2 days and mRNA levels of *Acp5* and *Atp6v0d2*were quantified by RT-qPCR. Data are presented as relative gene expression of gene of interest related to the reference gene *Hprt1*. *n* = 5 experiments. **D**–**E** Gene expression analysis of transcription factors *Nfatc1* (osteoclast) and *Irf8* (macrophage). Cells were stimulated with CM + RANKL (50 ng/ml) for 1 day and mRNA levels of *Nfatc1* and *Irf8* were quantified by RT-qPCR (**D**). Data are presented as relative gene expression of gene of interest related to the reference gene *Hprt1*. Ratio of *Nfatc1* to *Irf8* normalized expression levels was used as indicator for macrophage differentiation (**E**). *n* = 5 experiments. **F**–**G** Osteoclast formation of RANKL-primed osteoclast progenitor cells. Cells were stimulated with RANKL (50 ng/ml) for 2 days. On day 2, CM + RANKL were added. On day 5, cells were fixed, stained for TRAP and nuclei, and total numbers of formed OCs per well were counted. Osteoclasts were defined as TRAP-positive multinucleated giant cells with at least three nuclei. Overall osteoclast numbers (**F**) or osteoclast numbers divided into large (more than 30 nuclei per osteoclast), mid (between 15 and 30 nuclei per osteoclast) and small (less than 15 nuclei per osteoclast) (**G**) are shown. Data are presented as OC numbers per well. *n* = 4 experiments in duplicates (mean of duplicates was included in statistics). For all: Data are presented as mean + SD and single values are shown as dots. *p* values are calculated by ordinary one-way ANOVA with post hoc Bonferroni corrected multiple comparison. * is indicating significance against medium, + is indicating significance against RANKL, # is showing significance between respective planktonic and biofilm CM. * *p* < 0.05, ** *p* < 0.01, *** *p* < 0.001; + *p* < 0.05, +  + *p* < 0.01, +  +  + *p* < 0.001; # *p* < 0.05, ## *p* < 0.01, ### *p* < 0.001

**Fig. 2:**
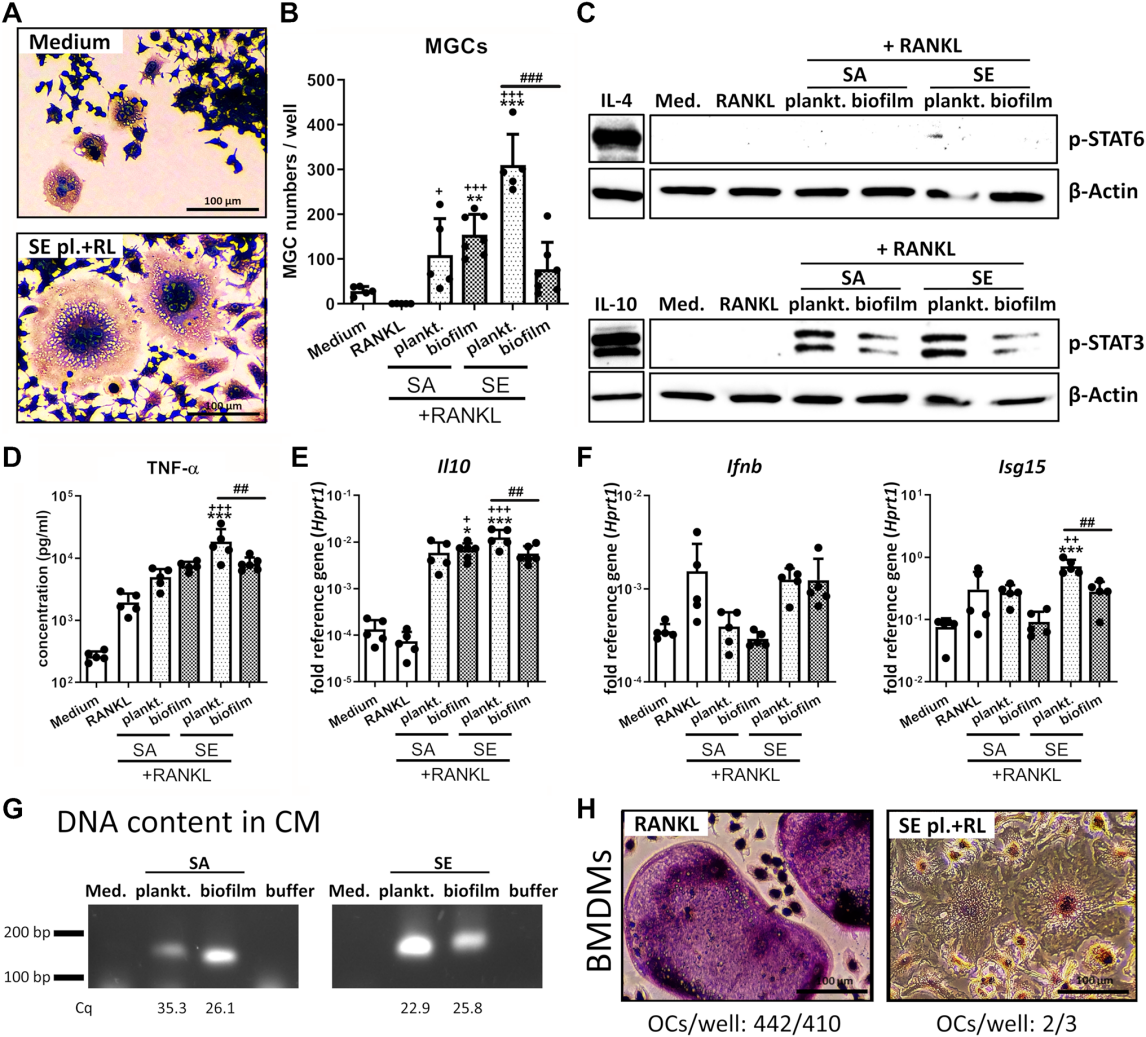
MGC formation and immune activation of macrophages upon stimulation with CM + RANKL. RAW 264.7 cells were cultivated in CM 1:1 diluted in fresh growth media (DMEM high glucose + 10% FCS + 1% Pen/Strep) and CM + RANKL-induced multinucleated giant cell (MGC) formation was investigated. **A**–**B** Cells were stimulated with CM + RANKL (50 ng/ml) for 5 days. On day 5, cells were fixed, stained for TRAP and nuclei, and MGCs per well were counted. **A** Visualization of formed MGCs. TRAP activity is shown by violet color development and nuclei are stained in blue. Upper picture is showing spontaneous MGC formation in medium control, lower picture is showing formed MGCs stimulated with RANKL + SE planktonic CM. **B** Total numbers of formed MGCs per well. MGCs were defined as low TRAP-positive multinucleated giant cells with at least three nuclei. Data are presented as MGC numbers per well. *n* = 5 experiments in duplicates (mean of duplicates was included in statistics). **C** Activation of STAT6 and STAT3 signaling in macrophages. Cells were stimulated with CM + RANKL (50 ng/ml) for 2 days and presence of phospho-STAT6 and phospho-STAT3 as activated forms of the transcription factors was visualized by western blot. IL-4 and IL-10 (both: 25 ng/ml for 1 h) were used as positive controls for respective STAT-pathway activation. β-Actin was used as loading control. *n* = 4 experiments. **D** Protein amounts of pro-inflammatory cytokine TNF-α in the supernatant. Cells were stimulated with CM + RANKL (50 ng/ml) for 2 days and protein concentration was quantified in the supernatant by multi cytokine bead array (CBA; LEGENDplex™). Data are presented as absolute concentration (pg/ml). *n* = 5 experiments. **E** Gene expression analysis of anti-inflammatory cytokine IL-10. Cells were stimulated with CM + RANKL (50 ng/ml) for 2 days and mRNA levels of *Il10* were quantified by RT-qPCR. Data are presented as relative gene expression of gene of interest related to the reference gene *Hprt1*. *n* = 5 experiments. **F** Gene expression analysis of IFN-β response. Cells were stimulated with CM + RANKL (50 ng/ml) for 1 day and mRNA levels of *Ifnb* and its target gene *Isg15* were quantified by RT-qPCR. Data are presented as relative gene expression of gene of interest related to the reference gene *Hprt1*. *n* = 5 experiments. **G** Free bacterial DNA content in CM. DNA was extracted from 1 ml CM and amplified by qPCR using SA-specific primers for gyrase B (product length: 147 bp) and SE-specific primers for gyrase A (product length: 194 bp). PCR products were visualized by agarose gel electrophoresis. Numbers below bands show the mean Cq value of *n* = 3 CM approaches. **H** Visualization of BMDM (bone marrow-derived macrophage)-formed osteoclasts or MGCs after 7 days of differentiation. BMDMs were stimulated with CM + RANKL (50 ng/ml) for 7 days. On day 7, cells were fixed, stained for TRAP and nuclei. Osteoclasts were defined as highly TRAP-positive and MGCs as low TRAP-positive multinucleated giant cells with at least three nuclei. TRAP activity is shown by violet color development and nuclei are stained in blue. Left picture shows osteoclast formation in RANKL control, right picture shows MGC formation after treatment with RANKL + SE planktonic CM. Number of counted osteoclasts per well is stated below the picture. *n* = 1 experiment in duplicates. For A, D + E: Data are presented as mean + SD and single values are shown as dots. p values are calculated by ordinary one-way ANOVA with post hoc Bonferroni corrected multiple comparison. * is indicating significance against medium, + is indicating significance against RANKL, # is showing significance between respective planktonic and biofilm CM. * *p* < 0.05, ** *p* < 0.01, *** *p* < 0.001; + *p* < 0.05, +  + *p* < 0.01, +  +  + *p* < 0.001; # *p* < 0.05, ## *p* < 0.01, ### *p* < 0.001

**Fig. 3:**
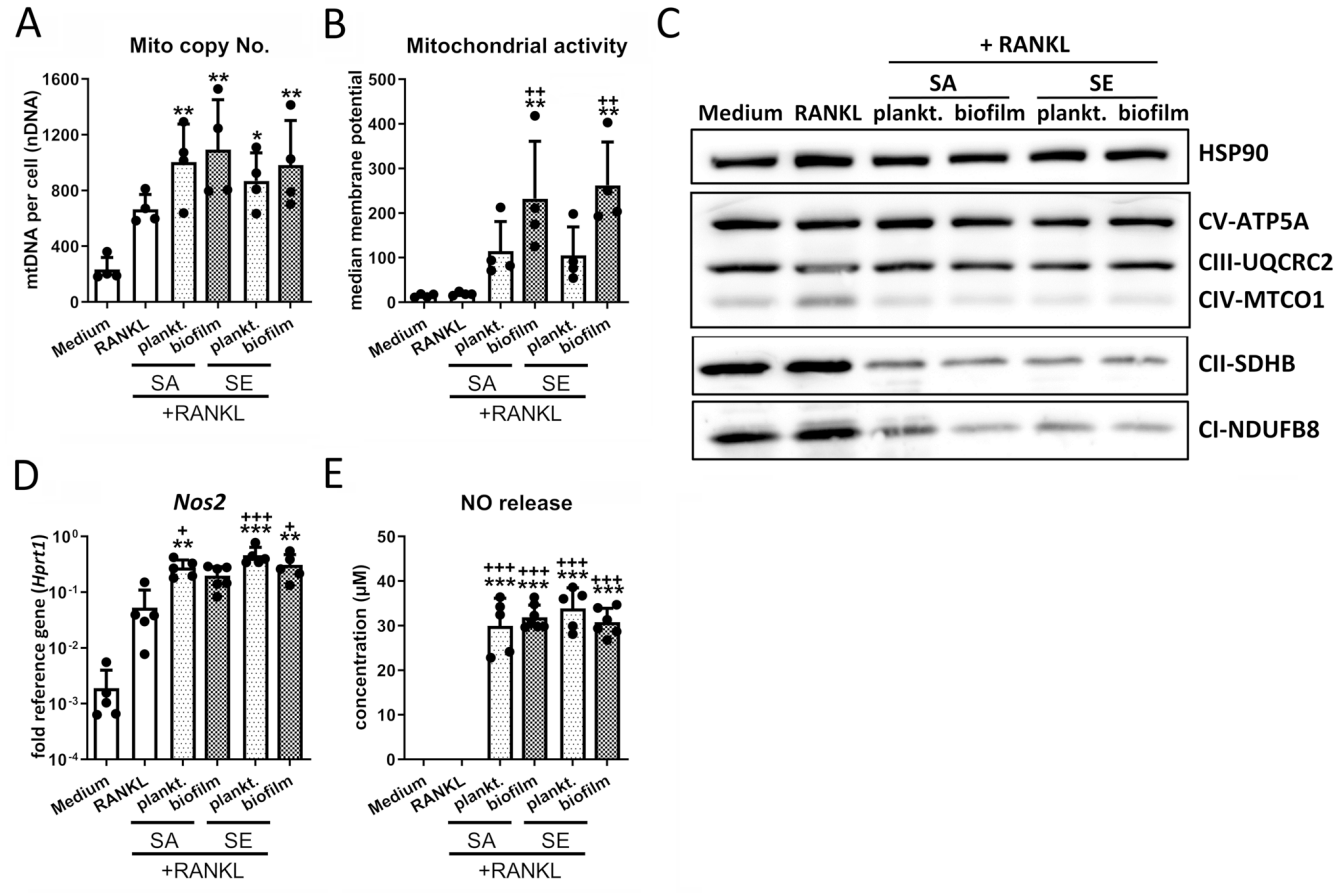
Metabolic changes during macrophage differentiation upon stimulation with CM + RANKL. RAW 264.7 cells were cultivated in CM 1:1 diluted in fresh growth media (DMEM high glucose + 10% FCS + 1% Pen/Strep) and effect of CM + RANKL stimulation on macrophage metabolism was investigated. **A** Number of mitochondria per macrophage. Cells were stimulated with CM + RANKL (50 ng/ml) for 4 days and mitochondria copy numbers were quantified by quantitative PCR. Data are presented as mitochondrial DNA (mtDNA) copies related to nuclear DNA (nDNA). *n* = 4 experiments. **B** Mitochondrial activity of macrophages. Cells were stimulated with CM + RANKL (50 ng/ml) for 4 days and mitochondrial activity was measured by FACS analysis using a membrane potential-dependent fluorescent dye. Data are presented as median mitochondrial potential measured by fluorescence intensity. *n* = 4 experiments. **C** Protein levels of OXPHOS complexes. Cells were stimulated with CM + RANKL (50 ng/ml) for 2 days and presence of OXPHOS complexes I-V was visualized by western blot. HSP-90 was used as loading control. *n* = 4 experiments. **D** Gene expression analysis of iNOS. Cells were stimulated with CM and RANKL (50 ng/ml) for 2 days and mRNA levels of *Nos2* were quantified by RT-qPCR. Data are presented as relative gene expression of gene of interest related to the reference gene *Hprt1*. *n* = 5 experiments. **E** NO release by macrophages. Cells were stimulated with CM + RANKL (50 ng/ml) for 2 days and NO content in the supernatant was quantified by Griess reaction. Data are presented as concentration in µM calculated by OD at 540 nm. *n* = 5 experiments. For A + B, D + E: Data are presented as mean + SD and single values are shown as dots. *p* values are calculated by ordinary one-way ANOVA with post hoc Bonferroni corrected multiple comparison. * is indicating significance against medium, + is indicating significance against RANKL, # is showing significance between respective planktonic and biofilm CM. * *p* < 0.05, ** *p* < 0.01, *** *p* < 0.001; + *p* < 0.05, +  + *p* < 0.01, +  +  + *p* < 0.001; # *p* < 0.05, ## *p* < 0.01, ### *p* < 0.001

The murine macrophage cell line RAW 264.7 (ATCC TIB-71, USA) was used for the experiments [33]. RAW 264.7 cells were cultivated in DMEM high glucose + 10% heat-inactivated FCS + 1% penicillin/streptomycin at 37 °C and 5% CO_2_. Before experiments, cells were harvested by scraping, centrifuged at 1300 rpm for 5 min, and resuspended in fresh growth media (DMEM high glucose + 10% FCS + 1% Pen/Strep). Cells then were transferred into suitable well plate formats at appropriate concentrations (for more information see Suppl. Table 1) and treated with CM 1:1 diluted in fresh cell growth media and recombinant mouse RANKL (50 ng/ml, bio-techne, UK). Initially, CM of day 3 and day 6 biofilms were evaluated separately. As they did not show differences, results were combined. This involved the experiments behind Fig. 1A–C, Fig. 2A–E, and Fig. 3C + D. In all other experiments, CM from day 6 biofilms were used. TLR ligands (Pam3CSK4: 25 ng/ml and CpG ODN 1668: 25 ng/ml, both InvivoGen, USA), recombinant murine cytokines (IL-10: 25 ng/ml, Peprotech, USA; TNF-α: 25 ng/ml, eBioscience, Germany or IFN-β: 0.2 and 2 ng/ml, BioLegend, USA), sodium L- or D-lactate (10, 15 and 20 mM, both Sigma-Aldrich, Germany) were added to the cells, respectively. For TLR-2 or TLR-9 inhibition, cells were pre-incubated with TLR-2 specific blocking antibody (10 µg/ml) or respective IgG isotype or TLR-9 inhibitory ODN 2088 (2 µM) or respective control ODN (all InvivoGen, USA) for 1 h in fresh growth media. IFN-β neutralization was done by simultaneously adding 50 U/ml anti-mouse IFN-β neutralizing antibody or 125 ng/ml of control IgG (both Invitrogen, USA). Fresh antibodies were added every 24 h. In experiments with incubation times longer than 2 days, 50 µM β-mercaptoethanol (PAN-Biotech GmbH, Germany) was added to reduce cell proliferation and avoid overgrowing of RAW 264.7 macrophages. Furthermore, in these experiments, half of the medium was exchanged late on day 2 or early on day 3 and, if necessary, on day 5 and cells were re-stimulated with half of stimuli. Experiments using RANKL-primed osteoclast progenitor cells were performed by treating the cells for 2 days with RANKL only followed by the addition of further stimuli together with the replacement of medium and RANKL on day 2.

To validate the results regarding the effects of CM stimulation on RANKL-induced osteoclastogenesis of RAW 264.7 cells, we repeated osteoclast formation in primary mouse bone marrow-derived macrophages (BMDMs). Bone marrow was flushed out of femora from adult C57BL/6 mice and cultivated in primary macrophage media (DMEM high glucose + 10% FCS + 1% Pen/Strep + 50 µM beta-mercaptoethanol) with 10–30% GM-CSF and M-CSF enriched L929 supernatants for 7 days to generate BMDMs. For osteoclast formation, 2*10^5^ cells were seeded in 500 µl primary macrophage media in a 24 well plate and cells were stimulated with 25 ng/ml M-CSF (bio-techne, UK) and 50 ng/ml RANKL together with 500 µl CM. A negative control with only M-CSF and a positive control with M-CSF and RANKL were included. Cells were re-stimulated by a half media exchange on day 3 and cell differentiation was evaluated microscopically on day 7.

### Tartrate-resistant acid phosphatase (TRAP) staining

TRAP staining of formed osteoclasts was performed after 5 or 6 days of stimulation: Media were removed and cells were fixed with fixation solution (26% v/v citrate solution 27 mM pH 3.6, 66% v/v acetone, 8% v/v 37% formaldehyde) for 30 s at RT. Cells were washed with ddH_2_O and 400 µl pre-warmed staining solution (1% v/v naphthol AS-BI phosphoric acid solution, 2% v/v tartrate solution, 4% v/v acetate solution 2.5 M pH 5.2 and 2% v/v diazotated Fast Garnet solution (1:1 sodium nitrite solution and Fast Garnet GBC Base solution, prepared in advance) in ddH_2_O, from Acid Phosphatase Kit, Sigma-Aldrich, Germany) was added per well and incubated at 37 °C for 30–60 min. Cells were washed with ddH_2_O and nuclei were counterstained with hematoxylin solution (Gill No. 3, Kit component) for 1 min. After removing hematoxylin solution, nuclei stain was developed with tap water for 10 min. Cells were washed with ddH_2_O and dried with open lid overnight. Osteoclast (OC) and inflammation-associated multinucleated giant cell (MGC) formation was evaluated by counting the cells (10 × magnification) using a cell culture microscope. OCs were defined as large TRAP-positive multinucleated cells (> 3 nuclei) with plain cell borders and clear cytosol, MGCs were defined as low TRAP-positive multinucleated cells (> 3 nuclei) having a more diffuse cell shape including granules. Pictures were taken with the Rebel microscope (Echo, USA).

### Gene expression analysis

Gene expression analysis of RAW 264.7 cells was performed after 1 or 2 days of stimulation. Supernatants and cells were frozen separately and stored at − 80 °C until further processing. Total RNA extraction was performed using the innuPREP RNA Mini Kit 2.0 (Analytik Jena, Germany) according to the manufacture’s protocol. In short, cells were scraped in lysis buffer and transferred to a DNA elution column. RNA in the lysate was precipitated by adding 70% ethanol, transferred to an RNA column, washed and eluted in RNAse free H_2_O. Total RNA concentration was measured using the NanoDrop^®^ ND-1000 spectrophotometer (Thermo Scientific, Germany). 1 µg of total RNA was subjected to cDNA synthesis using the Biozym cDNA synthesis Kit (Biozym Scientific GmbH, Germany) according to the manufacturer’s protocol using Oligo (dT) primer. For investigation of TLR-2/-9 inhibition, RNA was extracted by the ExtractMe total RNA Micro Spin Kit (BLIRT S.A., Gdańsk, Poland) and 1 µg total RNA was transcribed into cDNA by the enzymatics M-MuLV RT (Qiagen, MA–USA) according to the manufacturer’s protocols. A noRT sample (w/o Reverse Transcriptase) consisting of pooled total RNA of all samples of one experiment was prepared. cDNA was diluted 1:1 in H_2_O and stored at − 20 °C. 2 µl cDNA template and 400 nM of the respective primer pairs (Table 1) were used in qPCR. mRNA levels were evaluated in a two-step PCR reaction (StepOnePlus Real-Time PCR Cycler, Applied Biosystems, USA) with 60 °C annealing/extension temperature for 40 cycles using the 2 × qPCRBIO SyGreen Mix Hi-ROX (PCR Biosystems Ltd., UK). Quality of qPCR runs and specificity of qPCR products were controlled by melting curve comparison with included noRT and water samples for each experiment and primer pair and melting curve comparison. mRNA levels of the respective genes of interest (Table 1) were normalized to the reference gene *Hprt1* and calculated by the 2^−∆Cq^ method.

**Table 1:** List of oligonucleotides used for quantitative RT-PCR analysis

Gene	RefSeq	Forward primer	Reverse primer
*Acp5*	NM_001102405.1	TTCCAGGAGACCTTTGAGGA	GGTAGTAAGGGCTGGGGAAG
*Atp6v0d2*	NM_175406.3	TCAGATCTCTTCAAGGCTGTGCTG	GTGCCAAATGAGTTCAGAGTGATG
*Hprt1*	NM_013556.2	GGGGACATAAAAGTTATTGGTGG	CATTTTGGGGCTGTACTGCT
*Ifnb*	NM_010510.1	TGGGAGATGTCCTCAACTGC	CCAGGCGTAGCTGTTGTACT
*Il10*	NM_010548.2	GGTTGCCAAGCCTTATCGGA	ACCTGCTCCACTGCCTTGCT
*Irf8*	NM_001301811.1	CAGATCCTCCCTGACTGGTG	GCTTGCCCCCGTAGTAGAAG
*Isg15*	NM_015783.3	CCTGGTGAGGAACGAAAGGG	AAGCGTGTCTACAGTCTGCG
*Nfatc1*	NM_016791.4	CAGGGCTCACTATGAGACGG	AGCTGTAGCGTGAGAGGT
*Nos2*	NM_010927.4	CATGAGCTTGGTGTTTGGGTG	TCCGCAAATGTAGAGGTGGC
*Tnfa*	NM_013693.3	AAAATTCGAGTGACAAGCCTGTAG	CCCTTGAAGAGAACCTGGGAGTAG

Mouse specific primers were designed intron-flanking and included all transcript variants if possible and were obtained from biomers.net GmbH, Germany. If more transcript variants are present, RefSeq is given for transcript variant 1

### Cytometric bead array

Supernatants of day 2 gene expression analysis were used for cytometric bead array (CBA, LEGENDplex™, BioLegend, USA) according to the manufacturer’s protocol. A Mouse Inflammation Panel (Mix and Match Subpanel) was used including TNF-α. In short, supernatants were centrifuged, diluted 1:10 with Assay Buffer, standard samples were prepared and transferred into a V-bottom plate. Bead mix was prepared, added to the samples, and incubated on a shaker over night at 4 °C in the dark. Plates were then washed twice and incubated with the detection antibody for 1 h at RT while shaking. Streptavidin–phycoerythrin (SA–PE) was added and further incubated for 30 min at RT while shaking. Plates were washed twice before re-suspending the bead pellets in wash buffer. Data acquisition was done with a BD^®^ LSR II Flow Cytometer. Analysis and calculation of cytokine concentrations were performed with the included LEGENDplex™ Data Analysis Software (version 8.0).

### Immunoblotting

Protein analysis by Western blot was performed after 2 days of stimulation. RAW 264.7 cells were lysed in RIPA buffer (1% v/v NP-40 (IGEPAL^®^ CA-630), 0.25% sodium deoxycholate, 50 mM Tris pH 8.0, 150 mM NaCl, 1 mM EDTA pH 8.0, 1 mM Na_3_VO_4_) with EDTA-free protease inhibitors (cOmplete™ Tablets) and phosphatase inhibitors (PhosSTOP™, both Roche Diagnostics GmbH, Germany) for 1 h at 4 °C under rotation. Lysates were centrifuged at 14,000 rpm for 20 min at 4 °C, supernatants were transferred into fresh 1.5 ml reaction tubes and stored at − 80 °C. Protein concentration was determined by BCA assay (Cyanagen Srl, Italy), samples were adjusted to 10 µg protein per 20 µl with ddH_2_O and 5 µl 4 × SDS sample buffer with 10% β-mercaptoethanol, shaken for 2 min at 95 °C and stored at − 20 °C. For visualization of OXPHOS complexes, protein samples were not heated. 10 µg protein was loaded on pre-cast gradient 4–20% Tris–glycine gels (anamed Elektrophorese GmbH, Germany) and separated at 120 Volt. 5 µl of broad range, color pre-stained protein standard (New England Biolabs GmbH, Germany) was included on each gel. Proteins were transferred onto an Amersham™ Protran™ 0.45 µm nitrocellulose membrane (GE Healthcare, UK) covered in 4 + 4 Whatman papers (GE Healthcare, UK) soaked in transfer buffer (192 mM glycine, 25 mM Tris, 2.6 mM SDS, 0.5 mM Na_3_VO_4_, 15% v/v methanol) for 1 h at 2 mA/cm^2^. Membranes were stained with Ponceau S stain (0.5% Ponceau S, 3% trichloroacetic acid, 96.5% ddH_2_O) for 1 min to visualize transferred proteins. Blocking was done in BlueBlock PF (Serva Electrophoresis GmbH, Germany) for 30 min at RT with continuous shaking. Membranes were incubated with primary antibodies (Table 2) diluted in BlueBlock PF over night at 4 °C. Next day, membranes were incubated with the respective HRP-linked secondary antibody (Table 2) for 1 h at RT under shaking. Blots were developed with ECL substrate (WESTAR ETA C ULTRA 2.0, Cyanagen Srl, Italy) and developed with a ChemoStar ECL & Fluorescence Imager (Intas Science Imaging Instruments GmbH, Germany).

**Table 2:** List of antibodies used for immunoblotting (Western blot)

Protein	Source	Size (kDa)	Dilution	Company
β-Actin	Rabbit	42	1:1000	Proteintech, USA
HSP-90	Rabbit	90	1:1000	Cell Signaling Technology, USA
Phospho-STAT3 (Tyr705)	Rabbit	79, 86	1:1000	Cell Signaling Technology, USA
Phospho-STAT6 (Tyr641)	Rabbit	110	1:1000	Cell Signaling Technology, USA
Total OXPHOS (CI-V)	Mouse	20, 30, 40, 48, 55	1:1000	Abcam, UK
Anti-mouse IgG, HRP-linked	Horse		1:1000	Cell Signaling Technology, USA
Anti-rabbit IgG, HRP-linked	Goat		1:1000	Cell Signaling Technology, USA

Antibodies were all recommended for use in mouse and applied according to the manufacturer’s advice. Proteins were detected by chemiluminescent luminol reaction after incubation with respective HRP-linked secondary antibody and imaged in a ChemoStar ECL Imager

### Mitochondrial activity

Mitochondrial activity was measured after 4 days of stimulation. 100 nM of a mitochondrial membrane potential-sensitive dye (stock conc.: 1 mM in DMSO, MitoTracker^®^ Deep Red FM, Cell Signaling Technology, USA) was added to the cells for 30 min at 37 °C and 5% CO_2_. Cells were washed three times with cold PBS, scraped in PBS and transferred into FACS tubes. Mitochondrial activity was analyzed with a BD FACSCanto™ Flow Cytometer according to the fluorescence emission of the dye. Only the living cell population was included in the analysis using the Flowing Software (version 2.5.1, Turku Bioscience, Finland).

### Mitochondrial DNA copy number (Mito copy No.)

Mito copy No., which serves as a relative measure of mitochondrial DNA copies per cell, was evaluated in RAW 264.7 cells after 4 days of stimulation: DNA was extracted using the innuPREP DNA Mini Kit (Analytik Jena, Germany) according to the manufacturer’s protocol. In short, cells were lysed in lysis solution containing Proteinase K and DNA was extracted by a DNA binding column. DNA concentration of the eluate was measured using the NanoDrop^®^ ND-1000 spectrophotometer. 10 ng DNA was transferred in a qPCR reaction with 60 °C annealing/extension temperature for 40 cycles. Primer pairs specific for murine nuclear DNA (nDNA: B2M fw ATGGGAAGCCGAACATACTG, B2M rev CAGTCTCAGTGGGGGTGAAT, NC_000068.7) and mitochondrial DNA (mtDNA: fw CTAGAAACCCCGAAACCAAA, rev CCAGCTATCACCAAGCTCGT, NC_005089.1) were extracted from [34] and used for amplification of specific DNA products with 2 × qPCRBIO SyGreen Mix Hi-ROX. Specificity of products was controlled by water sample and melting curves. Mito copy No. was calculated according to formula: mtDNA per cell (nDNA) = 2 × 2^∆Cq^, ∆Cq = (nDNA Cq – mtDNA Cq).

### Nitric oxide (NO) detection

NO production was measured after 2 days of stimulation: Supernatants were directly used for NO detection in Griess assay. 50 µl sample as well as 50 µl of sodium nitrite (NaNO_2_) standard diluted in media (100–2.5 µM) and blank were transferred into half-area 96 well plates in duplicates. 50 µl of Griess reagent (1:1 solution A + B, mixed before use; A: 1% sulfonamide, 5% v/v H_3_PO_4_ in ddH_2_O; B: 0.1% N-1-naphthylethylenediamine dihydrochloride (NED) in ddH_2_O) was added and color reaction was determined at OD 540 / 620 nm in an absorbance microplate reader (Sunrise™, Tecan Trading AG, Switzerland). NO concentrations of samples were calculated by standard curve with the Tecan’s Magellan™ software.

### Statistical analysis

Experiments were done in *n* = 2–5 independent replicates as stated in the figure legends. Data are presented as mean + SD and single values as dots. Statistical evaluation was performed using ordinary one-way ANOVA with post hoc multiple comparison testing and Bonferroni correction. A p value below 0.05 was considered as statistically significant. * is indicating significance against medium, + is indicating significance against RANKL, $ is indicating significance against respective controls, # is showing significance between different treatments. Data analysis was performed with GraphPad Prism for Windows (Version 9.3.1, GraphPad Software Inc., USA).

## Results

### Simultaneous stimulation with CM suppresses RANKL-induced osteoclastogenesis and favors inflammation-associated MGC formation

First, we investigated the ability of RAW 264.7 macrophages to perform osteoclast differentiation in the presence of RANKL and the different CM. As expected, stimulation of macrophages with RANKL induced osteoclastogenesis, while the combined treatment with RANKL and CM significantly inhibited osteoclast formation (Fig. 1A). The few osteoclasts that developed despite CM treatment were smaller and rather isolated compared to the osteoclast clusters formed with RANKL alone (Fig. 1B). Impaired osteoclastogenesis was accompanied by a reduced induction of the osteoclast marker genes, tartrate-resistant acid phosphatase (TRAP, *Acp5*) and ATPase H + transporting V0 subunit D2 (ATP6V0D2) (Fig. 1C). Downregulation of the immune cell transcription factor IRF8 upon RANKL stimulation is crucial for activation and auto-upregulation of the osteoclast master regulator NFATc1 which subsequently initiates osteoclastogenic differentiation [12]. However, this did not occur in the samples after simultaneous treatment with RANKL and CM (Fig. 1D + E). Pre-incubation of macrophages with RANKL and subsequent CM at the stage of RANKL-primed osteoclast progenitor cells enabled osteoclast formation. Here, the numbers of osteoclasts were lowest for SE biofilm CM (Fig. 1F). By separately evaluating large, medium, and small osteoclasts, it became apparent that the various CM had different effects on osteoclast maturation (Fig. 1G). SA planktonic CM and to a lesser extend SA biofilm CM significantly limited the formation of large osteoclasts. Instead, SE planktonic CM showed similar osteoclast numbers as the RANKL control, whereas SE biofilm CM resulted in a decrease of osteoclast growth (Fig. 1G).

Although simultaneous stimulation with RANKL and CM inhibited osteoclast formation, the generation of another subtype of multinucleated giant cells through macrophage fusion was observed. In a pathological context not related to osteoclast formation, MGC formation is associated with chronic inflammation and an insufficient macrophage immune response [35, 36]. These inflammation-associated MGCs could be clearly distinguished from OCs, as they contained granules and displayed an activated morphology with filopodia (Fig. 2A). Macrophage fusion also occurred spontaneously in the control (medium alone), but the presence of CM led to the formation of large MGCs with multiple nuclei localized in the center of the cell. These cells were most prominently found in SE planktonic CM (Fig. 2B). Formation of inflammation-associated MGCs is associated with the activation of the IL-4/phospho-STAT6 pathway [37, 38]. Figure 2C shows that CM stimulation did not activate STAT6 but instead caused STAT3 tyrosine phosphorylation. Phospho-STAT3 levels were higher in planktonic CM compared to biofilm CM of both, SA and SE (Fig. 2C). TNF-α release was increased for all treatment conditions with the highest concentration detected in the supernatant of macrophages stimulated with SE planktonic CM (Fig. 2D). Accordingly, mRNA levels of the anti-inflammatory cytokine IL-10 were increased for all samples with the highest induction found upon stimulation with SE planktonic CM (Fig. 2E). In a previous study, we showed that planktonic CM of SA and SE induced *Ifnb* gene expression in macrophages [28]. Here, only in SE planktonic CM, the simultaneous stimulation with RANKL and CM induced expression of *Ifnb* and its target gene *Isg15* (Fig. 2F). Interestingly, we found that the content of free bacterial DNA in the CM correlated with inflammation-associated MGC formation and cytokine induction and the highest amounts of DNA were detected in SE planktonic CM (Fig. 2G). The decrease in osteoclast formation and corresponding increase in inflammation-associated MGC formation upon simultaneous stimulation with RANKL and SE planktonic CM were also confirmed using primary mouse BMDMs (Fig. 2H) which indicates that these findings are not a cell line artefact.

### Macrophage immune metabolism shifts toward increased mitochondrial contribution, while levels of electron transport chain (ETC) complexes are reduced by CM

We further analyzed the cellular metabolic activity upon simultaneous CM andRANKL stimulation. We observed an increase in mitochondrial DNA copy numbers (Mito copy No.) for all treatment conditions compared to the macrophage state; however, the increase was more pronounced after stimulation with CM and RANKL compared to RANKL (Fig. 3A). Correspondingly, RANKL alone did not alter the mitochondrial activity compared to the macrophage control, while in RANKL- and CM-treated cells, the mitochondrial activity was increased, particularly in the biofilm CM (Fig. 3B). Despite the observed increase in relative mitochondria numbers and mitochondrial activity, protein levels of ETC complexes were decreased in CM and RANKL-treated macrophages, in particular the small ETC complexes CI and CII (Fig. 3C). Our results indicate that although macrophages still exhibit a pro-inflammatory phenotype, generally associated with glycolytic activity, the cells increase their mitochondrial biomass and activity upon stimulation with RANKL and CM. Unexpectedly, these changes in mitochondria are accompanied by a decline in protein levels of ETC complexes. Because nitric oxide can cause loss of ETC complexes [39], we further analyzed iNOS-mediated NO release by CM and RANKL-treated macrophages. Gene expression of *Nos2* was induced by RANKL as previously described [40], but the increase was indeed more pronounced in the presence of additional CM (Fig. 3D). In contrast to *Nos2* mRNA levels, NO release was only induced when cells were treated in combination with CM (Fig. 3E). This suggests that NO indeed might contribute to the decline in the levels of ETC complexes in the CM-treated macrophages.

### CM effects on macrophage differentiation are rather mediated directly via TLR-2/-9 activation by bacterial molecules than by subsequent cytokine release

**Fig. 4:**
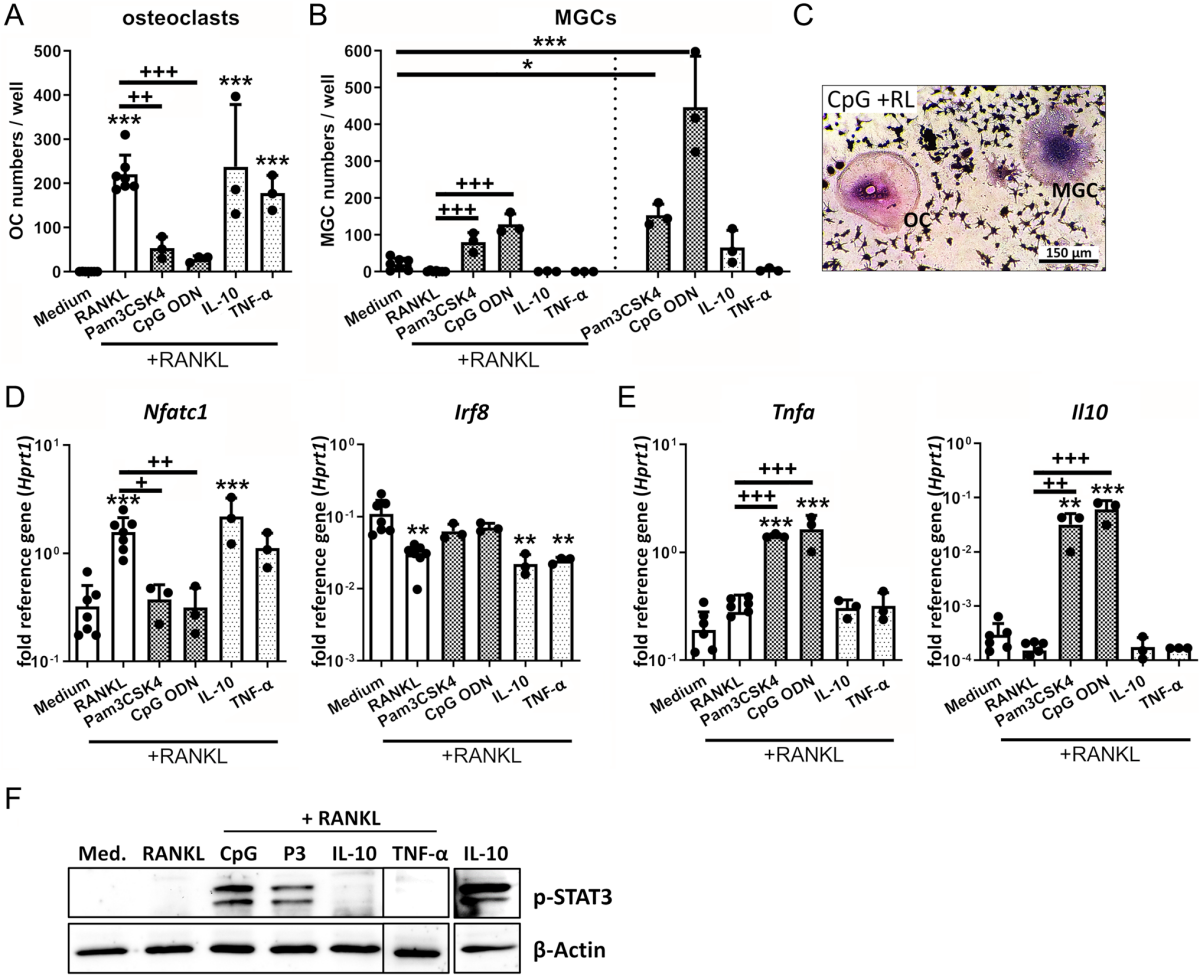
Effect of stimulation with TLR ligands or cytokines on osteoclast formation and immune activation of macrophages. RAW 264.7 cells were cultivated in growth media (DMEM high glucose + 10% FCS + 1% Pen/Strep) and effect of TLR ligands (TLR-2: Pam3CSK4, P3 1 µg/ml, TLR-9: CpG ODN 100 nM) or cytokines (IL-10 or TNF-α, both 25 ng/ml) ± RANKL stimulation on macrophage differentiation and immune activation was investigated. **A**–**B** Osteoclast and MGC formation of macrophages. Cells were stimulated with different stimuli ± RANKL (50 ng/ml) for 5 days. On day 5, cells were fixed, stained for TRAP and nuclei, and total numbers of formed osteoclasts (OCs) (**A**) or MGCs (**B**) per well were counted. Osteoclasts were defined as highly TRAP-positive and MGCs as low TRAP-positive multinucleated giant cells with at least three nuclei. Data are presented as OC / MGC numbers per well. *n* = 3 experiments in duplicates. **C** Morphological appearance of formed OC and MGC in TRAP/nuclei stain after stimulation with CpG ODN + RANKL (RL) for 5 days. **D**–**E** Gene expression analysis of transcription factors NFATc1 (osteoclast) or IRF8 (macrophage) (**D**) and pro-inflammatory cytokine TNF-α or anti-inflammatory cytokine IL-10 (**E**). Cells were stimulated with different stimuli and RANKL (50 ng/ml) for 2 days and mRNA levels of *Nfatc1*, *Irf8*, *Tnfa*, and *Il10* were quantified by RT-qPCR. Data are presented as relative gene expression of gene of interest related to the reference gene *Hprt1*. *n* = 3 experiments. F) Activation of STAT3 signaling in macrophages. Cells were stimulated with different stimuli and RANKL (50 ng/ml) for 2 days and presence of phospho-STAT3 as activated form of the transcription factor was visualized by western blot. IL-10 stimulation (25 ng/ml for 1 h) was used as positive control for STAT3-pathway activation. β-Actin was used as loading control. *n* = 3 experiments. For A + B, D + E: Data are presented as mean + SD and single values are shown as dots. p values are calculated by ordinary one-way ANOVA with post hoc Bonferroni corrected multiple comparison. * is indicating significance against medium, + is indicating significance against RANKL. * *p* < 0.05, ** *p* < 0.01, *** *p* < 0.001; + *p* < 0.05, +  + *p* < 0.01, +  +  + *p* < 0.001

To investigate whether the effect of CM on osteoclast and inflammation-associated MGC formation is mediated directly by bacterial mediators present in the CM or rather by subsequent cytokine release of CM-treated macrophages, we stimulated the cells either with the TLR-2 ligand Pam3CSK4, the TLR-9 ligand CpG ODN or the cytokines IL-10 and TNF-α, each in combination with RANKL (Fig. 4). TLR-2 and TLR-9 activation resulted in a significantly reduced RANKL-mediated OC formation (Fig. 4A) and significantly increased MGC numbers (Fig. 4B). However, TLR stimulation alone, especially when CpG was used (TLR-9), induced more MGCs than in combination with RANKL. The morphology of OCs and inflammation-associated MGCs could be clearly distinguished in the respective treatment groups where both cell types formed (Fig. 4C). In contrast to this, the cytokines IL-10 and TNF-α did not affect OC or MGC formation when added to RANKL (Fig. 4A + B). In line with these findings, RANKL-mediated induction of *Nfatc1* expression was diminished after stimulation with TLR ligands but not by the addition of cytokines. Accordingly, the downregulation of *Irf8* gene expression only occurred in RANKL or cytokine and RANKL-treated macrophages but not in the presence of TLR ligands (Fig. 4D). Furthermore, gene expression of *Tnfa* and *Il10* was only induced in TLR ligand / RANKL but not in cytokine / RANKL-stimulated macrophages (Fig. 4E). The induction of an immune response by TLR ligands was further reflected by the activation of the STAT3 pathway, which was only present upon TLR ligand and RANKL treatment (Fig. 4F).

**Fig. 5:**
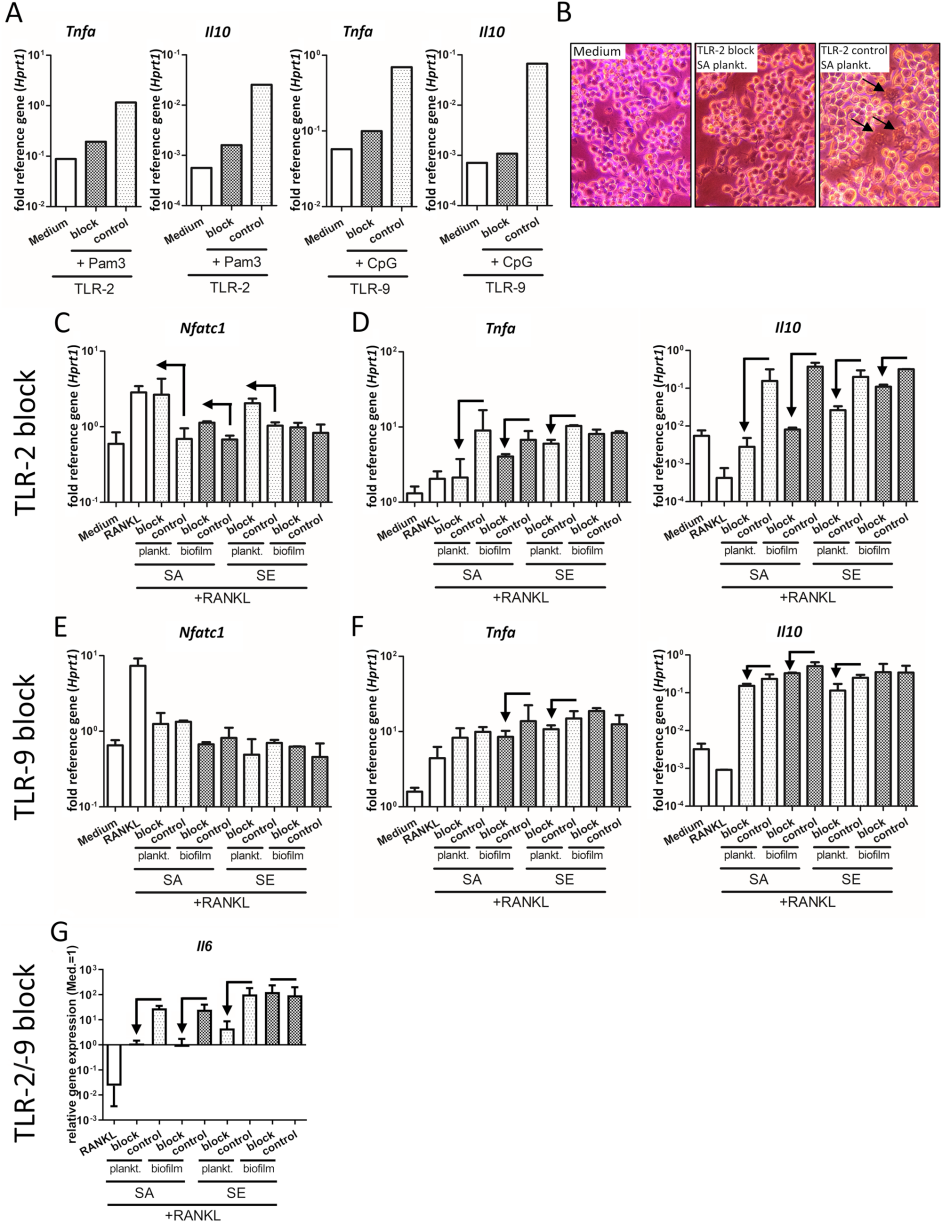
Effect of inhibited TLR-2 or TLR-9 activation on macrophage response toward CM. RAW 264.7 cells were pre-incubated with TLR-2-specific blocking antibody (10 µg/ml) or respective IgG isotype or TLR-9 inhibitory ODN 2088 (2 µM) or respective control ODN (all InvivoGen, USA) for 1 h in fresh growth media (DMEM high glucose + 10% FCS + 1% Pen/Strep). Cells were then stimulated by adding respective TLR ligands (TLR-2: 1 µg/ml Pam3CSK4, Pam3; TLR-9: 100 nM CpG ODN) or CM (same volume as fresh growth media; 1:1) + RANKL (50 ng/ml) and further blocking agents for 24 h. After stimulation time, cells were analyzed for gene expression of osteoclastogenesis marker NFATc1, pro-inflammatory cytokine TNF-α, and anti-inflammatory cytokine IL-10 by RT-qPCR. **A** Efficiency of TLR-2 or TLR-9 inhibition. Relative mRNA levels of cytokines *Tnfa* and *Il10* are shown after TLR-2 or TLR-9 blocking and stimulation by respective ligands for 24 h. One representative experiment is shown. **B** Morphology of cells after TLR-2 inhibition and 24 h stimulation with SA planktonic CM (left picture: unstimulated control, middle picture: TLR-2 blocking antibody and CM stimulation, right: isotype control and CM stimulation). Arrows highlight immune-activated macrophage phenotype, which was not present after TLR-2 inhibition. Magnification is 10X. **C**–**D** Relative mRNA levels of *Nfatc1* (**C**), *Tnfa*, or *Il10* (**D**) after TLR-2 blocking and stimulation by CM + RANKL for 24 h. **E + F** Relative mRNA levels of *Nfatc1* (**E**), *Tnfa* or *Il10* (**F**) after TLR-9 blocking and stimulation by CM + RANKL for 24 h. **G** Relative mRNA levels of *Il6* after combined TLR-2 / TLR-9 blocking and stimulation by CM + RANKL for 24 h. For C–G: *n* = 2 experiment, mean + SD are shown. Arrows indicate the effect of TLR blocking on the respective CM + RANKL treatment

Our results indicate that TLR-2 and TLR-9 activation might play a role in mediating the observed CM effects. As shown in Fig. 5 and Suppl. Fig. 1, inhibition of TLR-2 and/or TLR-9 signaling reduced the above described effects on macrophage immune activity and osteoclastogenesis. Thus, the observed CM-mediated effects are at least partially dependent on these pathways. Our data show that TLR-2 but not TLR-9 plays a crucial role in mediating the suppressive effects on RANKL-induced *Nfatc1* gene expression by planktonic CM (Fig. 5C + E). The induction of *Tnfa* and *Il10* gene expression by CM, however, seems to involve TLR-2 and TLR-9 activation (Fig. 5D + F). TLR-2 inhibition was more effective for planktonic CM and CM of SA, whereas the inhibition of TLR-9 signaling played a minor role and was only observed for SA biofilm and SE planktonic CM. As these two CM had the highest free DNA contents, the results indicate that free bacterial DNA might have contributed to the immune activation via TLR-9. However, combined inhibition of TLR-2 and TLR-9 displayed a pattern comparable to the inhibition of only TLR-2 signaling. This further indicates that TLR-2 activation is more relevant for the response against staphylococcal environments (Suppl.Fig. 1A + B). Interestingly, TLR-2 and TLR-9 signaling do not seem to be required for the anti-osteoclastogenic and pro-inflammatory effects of SE biofilm CM, as gene expression levels of *Nfatc1*, *Tnfa,* and the inflammation marker *Il6* (Fig. 5G) remained unaffected by the inhibition of the TLRs.

### IFN-β inhibits osteoclastogenesis dose dependently but seems not to be involved in the suppression of osteoclast formation upon stimulation with planktonic CM

**Fig. 6:**
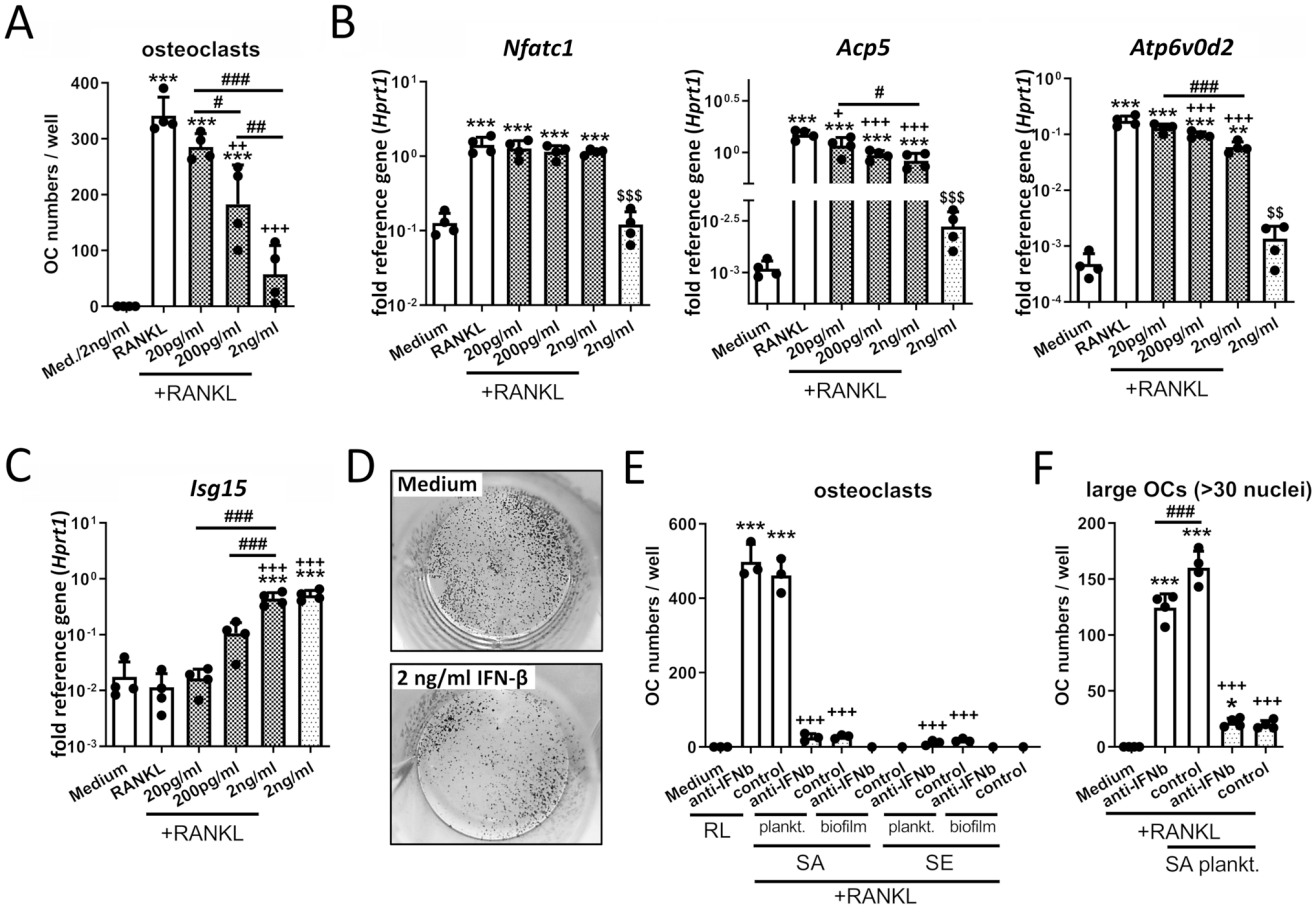
Effect of IFN-β on osteoclastogenic differentiation of macrophages. RAW 264.7 cells were cultivated in growth media (DMEM high glucose + 10% FCS + 1% Pen/Strep) ± CM and effect of recombinant IFN-β (**A**–**D**) or effect of IFN-β neutralization (E + F) on RANKL-mediated osteoclastogenesis of macrophages was analyzed. A) Osteoclast formation of macrophages. Cells were cultivated with IFN-β in different concentrations (20, 200 pg/ml and 2 ng/ml) + RANKL (50 ng/ml) for 5 days. On day 5, cells were fixed, stained for TRAP and nuclei, and total numbers of formed osteoclasts per well were counted. Osteoclasts (OCs) were defined as TRAP-positive multinucleated giant cells with at least three nuclei. Data are presented as OC numbers per well. *n* = 4 experiments in duplicates (mean of duplicates was included in statistics). **B**–**C** Expression analysis of osteoclastogenic marker genes (**B**) and IFN-β target gene *Isg15* (**C**). Cells were stimulated with different IFN-β concentrations + RANKL (50 ng/ml) for 2 days and mRNA levels of *Nfatc1*, *Acp5*, *Atp6v0d2* or *Isg15* were quantified by RT-qPCR. Data are presented as relative gene expression of gene of interest related to the reference gene *Hprt1*. *n* = 4 experiments. **D** Cell density after 5 days cultivation. Cells were cultivated with and without 2 ng/ml IFN-β for 5 days (controls of TRAP assay). On day 5, cells were fixed and stained for nuclei. Cells are presented as dark spots after hematoxylin stain. Representative pictures of *n* = 4 experiments in duplicates are shown. **E** Osteoclast formation of macrophages. Cells were stimulated with CM + RANKL (50 ng/ml) and IFN-β neutralizing antibody (50 U/ml) or IgG control for 5 days. On day 5, cells were fixed, stained for TRAP and nuclei, and total numbers of formed osteoclasts per well were counted. Osteoclasts were defined as TRAP-positive multinucleated giant cells with at least three nuclei. Data are presented as OC numbers per well. *n* = 3 experiments. Mean + SD are shown. **F** Osteoclast formation of RANKL-primed osteoclast progenitor cells. Cells were stimulated with RANKL (50 ng/ml) for 2 days. On day 2, SA plankt. CM + RANKL and IFN-β neutralizing antibody (50 U/ml) or IgG control were added. On day 5, cells were fixed, stained for TRAP and nuclei, and numbers of large osteoclasts per well were counted. Large osteoclasts were defined as TRAP-positive multinucleated giant cells with more than 30 nuclei. Data are presented as OC numbers per well. *n* = 2 experiments in duplicates. Mean + SD are shown. For A-C/E + F: Data are presented as mean + SD and single values are shown as dots. *p* values are calculated by ordinary one-way ANOVA with post hoc Bonferroni corrected multiple comparison. * is indicating significance against medium, + is indicating significance against RANKL, # is showing significance between different extracellular IFN-β concentrations or IFN-β antibody and control IgG. $ is indicating significance between 2 ng/ml IFN-β and 2 ng/ml IFN-β + RANKL. * *p* < 0.05, ** *p* < 0.01, *** *p* < 0.001; + *p* < 0.05, +  + *p* < 0.01, +  +  + *p* < 0.001; # *p* < 0.05, ## *p* < 0.01, ### *p* < 0.001; $ *p* < 0.05, $$ *p* < 0.01, $$$ *p* < 0.001

Since IFN-β may have a suppressive and regulatory effect on osteoclast formation [41, 42], we investigated whether IFN-β production by macrophages stimulated with planktonic CM contributed to the observed effects. For this purpose, macrophages were cultured in the presence of RANKL and increasing IFN-β concentrations. As shown in Fig. 6A, the addition of IFN-β resulted in a dose-dependent reduction of osteoclast formation. Although the induction of *Nfatc1* remained unaffected, the target genes *Acp5* and *Atp6v0d2* were significantly but not fully reduced. This indicates that IFN-β does not prevent RANKL-mediated osteoclastogenic gene induction (Fig. 6B). As a control, the mRNA level of the down-stream target of IFN-β signaling, interferon-stimulated gene 15 (*Isg15*), was determined and found to increase dose dependently (Fig. 6C). IFN-β is known to inhibit cell proliferation and indeed Fig. 6D shows that cell accumulation was reduced by the addition of IFN-β, indicating that the reduction of osteoclast formation is primarily depending on the inhibition of cell proliferation. To see if IFN-β signaling played a role in the observed reduction of osteoclast formation after CM treatment, IFN-β was inhibited with a neutralizing antibody. However, the inhibition of IFN-β signaling did not reduce the suppressive effect of the CM on osteoclastogenesis (Fig. 6E and Suppl. Fig. 2A + B). As shown in Fig. 1G, SA planktonic CM inhibited the maturation of RANKL-primed osteoclast progenitor cells. In a previous study, we showed that stimulation of macrophages with SA planktonic CM resulted in an IRF3-mediated IFN-β response [28]. Therefore, we investigated if IFN-β signaling was involved in this effect. We did not observe any differences in the maturation of large osteoclasts by RANKL-primed osteoclast progenitor cells upon IFN-β neutralization and stimulation with SA planktonic CM (Fig. 6F and Suppl. Fig. 2C + D). Thus, our data indicate that IFN-β is not involved in the inhibitory action of the CM on RANKL-induced osteoclast formation.

### High lactate concentration contributes to the suppressive effects of biofilm CM on osteoclastogenic differentiation of macrophages

**Fig. 7:**
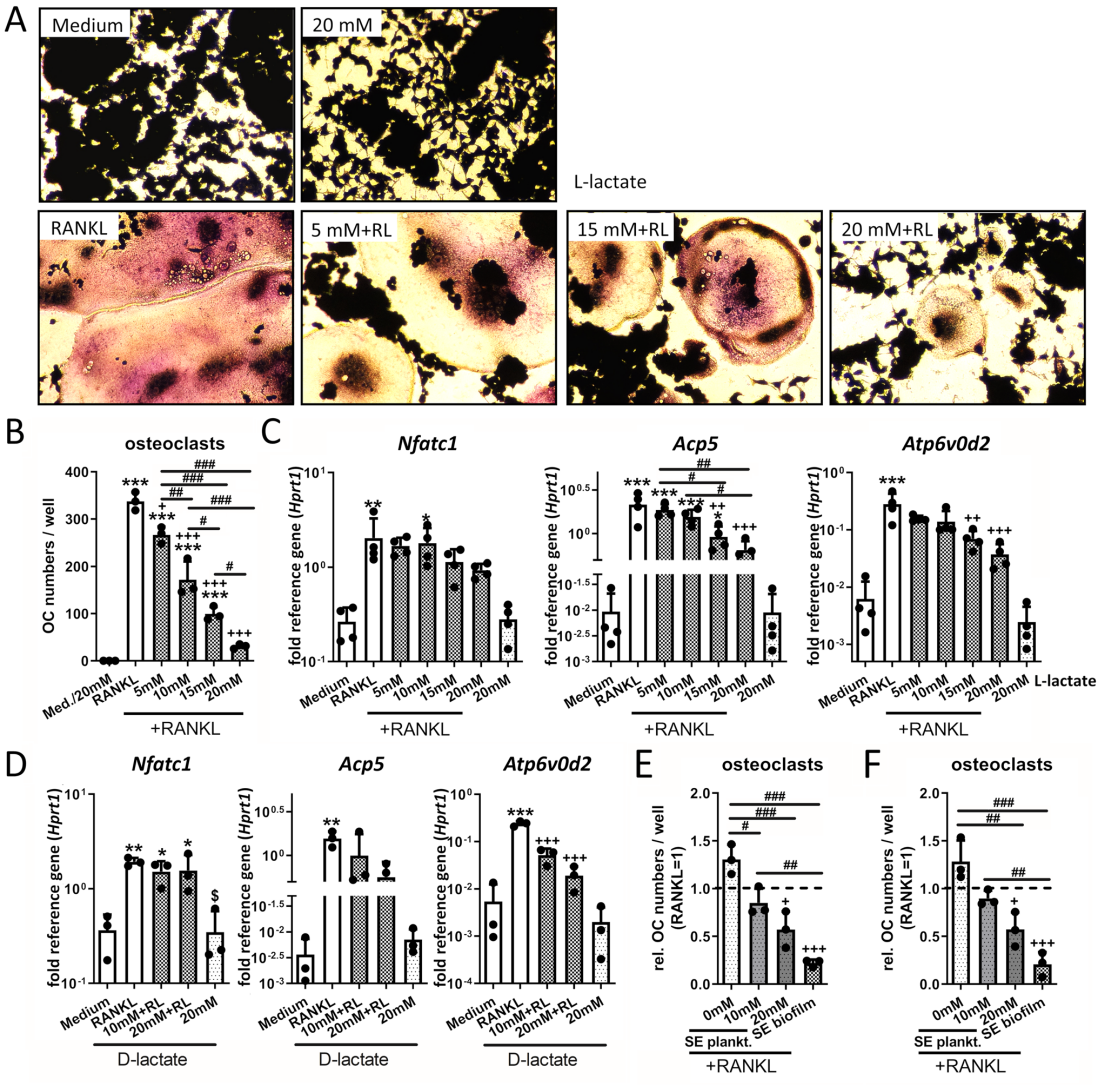
Effect of extracellular lactate on osteoclastogenic differentiation of macrophages. RAW 264.7 cells were cultivated in growth media (DMEM high glucose + 10% FCS + 1% Pen/Strep) ± CM and effect of extracellular L- or D-lactate on RANKL-mediated osteoclastogenesis of macrophages was investigated. **A**–**B** Osteoclast formation of macrophages in presence of L-lactate. Cells were cultivated with different concentrations of L-lactate (5, 10, 15 or 20 mM) ± RANKL (50 ng/ml) for 6 days. On day 6, cells were fixed, stained for TRAP and nuclei, and total numbers of formed osteoclasts (OCs) per well were counted. **A** Morphological appearance of formed OCs. **B** Osteoclast numbers per well. Osteoclasts were defined as TRAP-positive multinucleated giant cells with at least three nuclei. Data are presented as OC numbers per well. *n* = 3 experiments in duplicates (mean of duplicates was included in statistics). **C** Gene expression analysis of osteoclastogenesis marker genes. Cells were stimulated with different L-lactate concentrations and RANKL (50 ng/ml) for 2 days and mRNA levels of *Nfatc1*, *Acp5* or *Atp6v0d2* were quantified by RT-qPCR. Data are presented as relative gene expression of gene of interest related to the reference gene *Hprt1*. *n* = 4 experiments. **D** Gene expression analysis of osteoclastogenesis marker genes. Cells were stimulated with different D-lactate concentrations (10 and 20 mM) and RANKL (50 ng/ml) for 2 days and mRNA levels of *Nfatc1*, *Acp5* or *Atp6v0d2* were quantified by RT-qPCR. Data are presented as relative gene expression of gene of interest related to the reference gene *Hprt1*. *n* = 3 experiments. **E**–**F** Osteoclast formation of RANKL-primed osteoclast progenitor cells in presence of L- or D-lactate and stimulation with SE planktonic CM. Cells were stimulated with RANKL (50 ng/ml) for 2 days. On day 2, CM + RANKL and different concentrations of L-lactate (**E**) or D-lactate (**F**) (10 or 20 mM) were added. On day 5, cells were fixed, stained for TRAP and nuclei, and total numbers of formed osteoclasts per well were counted. Osteoclasts were defined as TRAP-positive multinucleated giant cells with at least three nuclei. Data are presented as relative OC numbers per well. *n* = 3 experiments in duplicates (mean of duplicates was included in statistics). For **B**–**F**: Data are presented as mean + SD and single values are shown as dots. *p* values are calculated by ordinary one-way ANOVA with post hoc Bonferroni corrected multiple comparison. * is indicating significance against Medium, + is indicating significance against RANKL, # is showing significance between different lactate concentrations or respective planktonic and biofilm CM. $ is indicating significance between 20 mM lactate and 20 mM lactate + RANKL. * *p* < 0.05, ** *p* < 0.01, *** *p* < 0.001; + *p* < 0.05, +  + *p* < 0.01, +  +  + *p* < 0.001; # *p* < 0.05, ## *p* < 0.01, ### *p* < 0.001; $ p < 0.05, $$ *p* < 0.01, $$$ *p* < 0.001

Bacterial biofilm environments are characterized by high levels of bacteria-derived lactate [23]. We had previously confirmed this metabolic profile for our biofilm CM as well [28]. To investigate if the lactate accumulation contributed to the suppressive effect of biofilm CM on osteoclastogenesis, we performed osteoclastogenic differentiation in the presence of high extracellular lactate levels. RAW macrophages were stimulated with RANKL and sodium L-lactate was added at increasing concentrations. Increased L-lactate concentrations led to a decrease in size and numbers of osteoclasts (Fig. 7A + B). mRNA levels of the transcription factor NFATc1 were slightly but non-significantly reduced, even at high L-lactate concentrations, whereas the expression of the NFATc1 target genes *Acp5* and *Atp6v0d2* decreased in a dose-dependent manner (Fig. 7C). SA and SE biofilms express the enzymes for L- and D-lactate production (Suppl. Fig. 3A + B) and thus, the biofilm CM most likely contain both stereoisomers. We, therefore, analyzed the effects of D-lactate on macrophage osteoclastogenesis. Again, extracellular addition of D-lactate suppressed osteoclastogenesis by reducing *Acp5* and *Atp6v0d2* expression, while *Nfatc1* mRNA levels remained unaffected (Fig. 7D). To evaluate if extracellular lactate is the cause of the reduced osteoclast formation observed for RANKL-primed osteoclast progenitor cells treated with SE biofilm CM (Fig. 1F), we stimulated these cells in combination with lactate and compared the osteoclast numbers. SE planktonic CM led to a slight but non-significant increase in osteoclast formation of RANKL-primed osteoclast progenitor cells compared to RANKL alone, whereas the addition of L- (Fig. 7E) or D-lactate (Fig. 7F) dose dependently decreased OC numbers; however, not to the same extent as SE biofilm CM. We, therefore, hypothesize that high lactate levels can contribute to the suppressive effect of biofilm CM on osteoclast formation, but are not sufficient to explain the observed reduction in osteoclast numbers.

## Discussion

Osteoclast-mediated bone degradation and subsequent implant loosening is a major consequence of chronic implant-related bone infections. As chronicity of these infections is associated with biofilm formation, we investigated if a biofilm environment had an impact on RANKL-mediated osteoclastogenesis of macrophages. Therefore, we generated CM from planktonic and biofilm cultures of SA and SE and co-stimulated RAW 264.7 macrophages with the CM and RANKL to mimic the inflammatory and osteoclastogenic environment of infected bone. The SA strain used in our study (UAMS-1) expresses certain virulence factors and is a rather moderate biofilm producer in vitro whereas the SE strain RP62A is associated with strong in vitro biofilm formation and low-grade inflammation. In our study, we found that all CM suppressed NFATc1 transcription factor activity and favored immune activation and MGC formation over RANKL-mediated osteoclastogenesis. This effect was strongest in SE planktonic CM. However, when CM were added to cells already primed into osteoclast progenitor cells through RANKL pre-incubation, the effects of CM treatment on osteoclastogenesis differed. Here, the highest OC numbers were found in SE planktonic CM, whereas reduced osteoclast formation was still observed for SE biofilm CM and both SA CM treatments. The effects of planktonic CM mainly relied on TLR activation and were independent of lactate levels, whereas in biofilm CM, high lactate levels additionally supported the observed effects.

The effect of TLR activation on osteoclast formation is dependent on the commitment stage of the precursor cells (reviewed in [22]): Stimulation of uncommitted macrophages with TLR ligands and RANKL inhibits osteoclastogenesis [43] whereas TLR activation of RANKL-primed osteoclast progenitor cells increases osteoclast maturation via inflammatory cytokines such as TNF-α [44, 45]. Our data show that TLR-2 and TLR-9 activation resulted in a suppression of RANKL-induced osteoclastogenesis when uncommitted macrophages were used. The inhibition of RANKL-induced osteoclastogenesis by TLR activation is described to depend on a decreased expression of the osteoclastogenic transcription factor NFATc1 and an increased expression of the macrophage lineage transcription factor IRF8 [46, 47]. In line with this, we show that the simultaneous stimulation of macrophages with RANKL and CM inhibited *Nfatc1* induction and, thus, also osteoclast differentiation whereas *Irf8* gene expression remained stable. Surprisingly, this did not only occur in the planktonic environment which is associated with a strong TLR activation but also in the biofilm CM. We found that TLR-2 but not TLR-9 was important for the suppression of RANKL-induced *Nfatc1* gene expression in the planktonic CM. However, this was less obvious for the biofilm CM, and thus could not explain the observed anti-osteoclastogenic effect in the biofilm environments. The fact that RANKL-primed osteoclast progenitor cells were inhibited by biofilm CM whereas SE planktonic CM supported osteoclast maturation underlines that there are different mechanisms behind the mode of action of planktonic and biofilm CM. In line with a study investigating SA infections on RANKL-primed bone marrow macrophages [48], we found that the SA planktonic environment exerted a suppressive effect on the maturation and fusion of large osteoclasts. This effect was not seen for SE planktonic CM which reflects the differences in virulence between SA and SE. Our results are, however, in contrast to a study showing that stimulation of RANKL-primed BMDMs with SA supernatants promotes osteoclastogenesis TLR dependently [49], indicating that there might also be variations between SA strains.

In a previous study, we showed that SA planktonic CM induced an IRF3-mediated IFN-β response in macrophages [28]. As IFN-β can antagonize RANKL-induced osteoclast formation [50] and plays a role in LPS-mediated suppression of osteoclastogenesis [51], we investigated the effect of IFN-β in SA and SE supernatants. In line with the literature [50, 52], the addition of extracellular IFN-β dose dependently suppressed RANKL-induced osteoclast formation. However, the inhibition of IFN-β signaling neither prevented CM effects on RANKL-mediated osteoclastogenesis of uncommitted macrophages nor explained the inhibitory effect of SA planktonic CM on osteoclast maturation of RANKL-primed osteoclast progenitor cells. Indeed, it was shown before that IFN-β only suppresses osteoclast formation of uncommitted bone marrow macrophages, i.e., when given directly at the onset of RANKL-induced osteoclastogenesis whereas addition of IFN-β to RANKL-primed pre-osteoclasts no longer exerted this effect [53]. In this study, we did not observe an induction of *Ifnb* gene expression upon simultaneous stimulation with SA planktonic CM and RANKL. Thus, it rather seemed that the previously detected IRF3-mediated IFN-β induction by SA planktonic CM alone was less pronounced in the presence of RANKL, and therefore did not play a role in the suppressive effects on osteoclast formation.

Simultaneous stimulation of macrophages with RANKL and CM favored macrophage immune activation indicated by increased TNF-α and IL-10 levels, activation of the STAT3 pathway, and formation of MGCs. These effects could partly be mimicked by stimulation with TLR-2 or -9 ligands in the presence of RANKL. Again, TLR-2 seemed to be the pre-dominant immunostimulatory pathway for SA planktonic CM but was also involved in the effects of SA biofilm and SE planktonic CM, where TLR-9 activation contributed to *Tnfa* and *Il10* induction. MGC formation and TNF-α release were most pronounced in SE planktonic CM where also the highest content of free bacterial DNA was found in the medium. As we could show that stimulation with the TLR-9 ligand CpG ODN highly induced MGC formation, we suggest that TLR-9 activation by free bacterial DNA is an important mechanism behind the pro-inflammatory and MGC inducing effects of SE planktonic CM. In vivo, MGCs are associated with chronic inflammation and an inadequate macrophage immune response that aims to locally restrict the infection. MGC formation is, for example, found in granuloma formation, foreign body reactions, and dental implant infections. However, their exact function is still not fully understood [35, 54]. To our knowledge, these cells have not yet been described for staphylococci infections and a potential contribution of MGCs in implant-related bone infections and associated biofilm formation should be further investigated. Interestingly, the effects of SE biofilm CM seemed to be independent of TLR-2 or -9 activation. This indicates that immunostimulatory bacterial molecules such as cell wall components or extracellular DNA are restrained by the solid SE biofilm matrix and, therefore, might not be present in the supernatant in sufficient quantity to be important for the immune response.

Recent studies showed that an increase in extracellular lactate concentrations has an impact on the metabolic activity of immune cells and reduces glycolytic activity while promoting mitochondrial oxidative phosphorylation (OXPHOS) or the polyol pathway [55, 56]. RANKL-mediated osteoclastogenesis is relying on both glycolysis and mitochondrial respiration. While increased glycolytic activity and subsequent lactate production are crucial for osteoclast formation and activity, ATP generation through mitochondrial OXPHOS is important for maintaining ATP levels during the energy consuming differentiation process [57–59]. As expected, we could show that RANKL stimulation increased the mitochondrial copy numbers per cell, but this was even further promoted by the addition of CM. Interestingly, mitochondrial activity was not altered in RANKL-treated cells. Addition of CM, however, increased mitochondrial activity, which was more pronounced for biofilm CM compared to planktonic CM treatments. Unexpectedly, we found the proteins of the electron transport chain to be decreased after co-stimulation with RANKL and CM. We hypothesize that this is due to the observed NO production which is part of the pro-inflammatory macrophage response [39, 60].

Our study shows that addition of extracellular lactate caused a dose-dependent decrease in RANKL-mediated osteoclast formation. A recent study had shown that glycolysis-derived lactate from macrophages during osteoclastogenic activity supported bone resorption, while the addition of extracellular lactate (5 mM) had no effect on the activity of already differentiated osteoclasts [24]. This indicates that just like for TLR stimulation [22], the effects of lactate might depend on the commitment stage of the macrophages, the timepoint of exposure but also on the source and concentration of the lactate. Addition of lactate to SE planktonic CM decreased osteoclast maturation of RANKL-primed osteoclast progenitor cells even though this did not reach the suppressive levels of SE biofilm CM. Thus, in a bacterial infection, high lactate concentrations in the local biofilm environment might indeed impair osteoclast formation and maturation. We suggest that these elevated lactate concentrations might interfere with the metabolic requirements of osteoclast differentiation.

In conclusion, our study shows that the bacterial environment of planktonic or biofilm staphylococci inhibits RANKL-induced osteoclastogenesis of uncommitted macrophages. TLR activation was less important in the biofilm environments than for planktonic environments. In biofilm CM, high lactate levels additionally contributed to the anti-osteoclastogenic effects not only on uncommitted macrophages but also on the maturation of RANKL-primed osteoclast progenitor cells. Osteoclastogenesis during chronic implant-related bone infections, therefore, seems to be mediated indirectly through the release of pro-inflammatory mediators from the macrophages. Accordingly, biofilm dispersal over time, the release of planktonic bacteria, and the (re-)induction of a pro-inflammatory immune response might be the pre-dominant phase of osteoclast-mediated bone destruction.

As planktonic bacteria are more vulnerable to antibiotics and immune cells, therapeutic-induced biofilm dispersion is a potential approach to control biofilm infections [61]. Furthermore, the success of immunotherapy in cancer encourages the use of such strategies to stimulate the immune response against biofilms [7]. Despite the enormous potential of these new treatment options, our study highlights that both strategies carry the risk of (re-)inducing an inflammation-associated increase in osteoclastogenic activity, either indirectly via release of planktonic bacteria or directly by immunomodulation. This should be considered when introducing such therapeutic approaches in implant-related bone infections.

## Supplementary Information

Below is the link to the electronic supplementary material.

Supplementary file1 (DOCX 697 KB)

## Data Availability

The raw data will be available on reasonable request.

## References

[1] Josse J, Valour F, Maali Y, Diot A, Batailler C, Ferry T (2019). Interaction between staphylococcal biofilm and bone: how does the presence of biofilm promote prosthesis loosening?. Front Microbiol.

[2] Wagner C, Hansch GM (2017). Mechanisms of bacterial colonization of implants and host response. Adv Exp Med Biol.

[3] Otto M (2018). Staphylococcal biofilms. Microbiol Spectr..

[4] Watters C, Fleming D, Bishop D, Rumbaugh KP (2016). Host responses to biofilm. Prog Mol Biol Transl Sci.

[5] Zimmerli W, Sendi P (2011). Pathogenesis of implant-associated infection: the role of the host. Semin Immunopathol.

[6] Arciola CR, Campoccia D, Montanaro L (2018). Implant infections: adhesion, biofilm formation and immune evasion. Nat Rev Microbiol.

[7] Seebach E, Kubatzky KF (2019). Chronic implant-related bone infections-can immune modulation be a therapeutic strategy?. Front Immunol.

[8] Gries CM, Kielian T (2017). Staphylococcal biofilms and immune polarization during prosthetic joint infection. J Am Acad Orthop Surg.

[9] Zimmerli W, Sendi P (2017). Orthopaedic biofilm infections. APMIS.

[10] Tande AJ, Patel R (2014). Prosthetic joint infection. Clin Microbiol Rev.

[11] Feng X, Teitelbaum SL (2013). Osteoclasts: new insights. Bone Res.

[12] Zhao B, Takami M, Yamada A, Wang X, Koga T, Hu X (2009). Interferon regulatory factor-8 regulates bone metabolism by suppressing osteoclastogenesis. Nat Med.

[13] Kubatzky KF, Uhle F, Eigenbrod T (2018). From macrophage to osteoclast - how metabolism determines function and activity. Cytokine.

[14] Xing L, Schwarz EM, Boyce BF (2005). Osteoclast precursors, Rankl/Rank, and immunology. Immunol Rev.

[15] Souza PP, Lerner UH (2013). The role of cytokines in inflammatory bone loss. Immunol Invest.

[16] Haynes DR (2004). Bone lysis and inflammation. Inflamm Res.

[17] Josse J, Velard F, Gangloff SC (2015). Staphylococcus aureus vs. Osteoblast: relationship and consequences in osteomyelitis. Front Cell Infect Microbiol.

[18] Wright JA, Nair SP (2010). Interaction of Staphylococci with bone. Int J Med Microbiol.

[19] Pietrocola G, Arciola CR, Rindi S, Di Poto A, Missineo A, Montanaro L (2011). Toll-like receptors (TLRs) in innate immune defense against Staphylococcus aureus. Int J Artif Organs.

[20] Mendoza Bertelli A, Delpino MV, Lattar S, Giai C, Llana MN, Sanjuan N (2016). Staphylococcus aureus protein A enhances osteoclastogenesis via TNFR1 and EGFR signaling. Biochim Biophys Acta.

[21] Wang Y, Liu X, Dou C, Cao Z, Liu C, Dong S (2017). Staphylococcal protein A promotes osteoclastogenesis through MAPK signaling during bone infection. J Cell Physiol.

[22] Souza PPC, Lerner UH (2019). Finding a toll on the route: the fate of osteoclast progenitors after toll-like receptor activation. Front Immunol.

[23] Yamada KJ, Kielian T (2019). Biofilm-leukocyte cross-talk: impact on immune polarization and immunometabolism. J Innate Immun.

[24] Taubmann J, Krishnacoumar B, Bohm C, Faas M, Muller DIH, Adam S (2020). Metabolic reprogramming of osteoclasts represents a therapeutic target during the treatment of osteoporosis. Sci Rep.

[25] Muthukrishnan G, Masters EA, Daiss JL, Schwarz EM (2019). Mechanisms of immune evasion and bone tissue colonization that make staphylococcus aureus the primary pathogen in osteomyelitis. Curr Osteoporos Rep.

[26] Le KY, Park MD, Otto M (2018). Immune evasion mechanisms of *Staphylococcus epidermidis* biofilm infection. Front Microbiol.

[27] Gillaspy AF, Hickmon SG, Skinner RA, Thomas JR, Nelson CL, Smeltzer MS (1995). Role of the accessory gene regulator (agr) in pathogenesis of staphylococcal osteomyelitis. Infect Immun.

[28] Seebach E, Elschner T, Kraus FV, Souto-Carneiro M, Kubatzky KF (2023). Bacterial and metabolic factors of staphylococcal planktonic and biofilm environments differentially regulate macrophage immune activation. Inflammation.

[29] Tomizawa T, Ishikawa M, Bello-Irizarry SN, de Mesy Bentley KL, Ito H, Kates SL (2020). Biofilm producing *Staphylococcus epidermidis* (RP62A Strain) inhibits osseous integration without osteolysis and histopathology in a murine septic implant model. J Orthop Res.

[30] Seebach E, Holschbach J, Buchta N, Bitsch RG, Kleinschmidt K, Richter W (2015). Mesenchymal stromal cell implantation for stimulation of long bone healing aggravates Staphylococcus aureus induced osteomyelitis. Acta Biomater.

[31] Christensen GD, Simpson WA, Younger JJ, Baddour LM, Barrett FF, Melton DM (1985). Adherence of coagulase-negative staphylococci to plastic tissue culture plates: a quantitative model for the adherence of Staphylococci to medical devices. J Clin Microbiol.

[32] Beenken KE, Blevins JS, Smeltzer MS (2003). Mutation of sarA in Staphylococcus aureus limits biofilm formation. Infect Immun.

[33] Raschke WC, Baird S, Ralph P, Nakoinz I (1978). Functional macrophage cell lines transformed by abelson leukemia virus. Cell.

[34] Malik AN, Czajka A, Cunningham P (2016). Accurate quantification of mouse mitochondrial DNA without co-amplification of nuclear mitochondrial insertion sequences. Mitochondrion.

[35] Ahmadzadeh K, Vanoppen M, Rose CD, Matthys P, Wouters CH (2022). Multinucleated giant cells: current insights in phenotype, biological activities, and mechanism of formation. Front Cell Dev Biol..

[36] Brooks PJ, Glogauer M, McCulloch CA (2019). An Overview of the derivation and function of multinucleated giant cells and their role in pathologic processes. Am J Pathol.

[37] Helming L, Gordon S (2009). Molecular mediators of macrophage fusion. Trends Cell Biol.

[38] Pereira M, Petretto E, Gordon S, Bassett JHD, Williams GR, Behmoaras J (2018). Common signalling pathways in macrophage and osteoclast multinucleation. J Cell Sci.

[39] Palmieri EM, Gonzalez-Cotto M, Baseler WA, Davies LC, Ghesquiere B, Maio N (2020). Nitric oxide orchestrates metabolic rewiring in M1 macrophages by targeting aconitase 2 and pyruvate dehydrogenase. Nat Commun.

[40] Huang R, Wang X, Zhou Y, Xiao Y (2017). RANKL-induced M1 macrophages are involved in bone formation. Bone Res.

[41] Feng X (2005). RANKing intracellular signaling in osteoclasts. IUBMB Life.

[42] Xiong Q, Zhang L, Ge W, Tang P (2016). The roles of interferons in osteoclasts and osteoclastogenesis. Joint Bone Spine.

[43] Takami M, Kim N, Rho J, Choi Y (2002). Stimulation by toll-like receptors inhibits osteoclast differentiation. J Immunol.

[44] Kim J, Yang J, Park OJ, Kang SS, Kim WS, Kurokawa K (2013). Lipoproteins are an important bacterial component responsible for bone destruction through the induction of osteoclast differentiation and activation. J Bone Miner Res.

[45] Zou W, Schwartz H, Endres S, Hartmann G, Bar-Shavit Z (2002). CpG oligonucleotides: novel regulators of osteoclast differentiation. FASEB J.

[46] Chen Z, Su L, Xu Q, Katz J, Michalek SM, Fan M (2015). IL-1R/TLR2 through MyD88 divergently modulates Osteoclastogenesis through regulation of nuclear factor of activated *T* cells c1 (NFATc1) and B lymphocyte-induced maturation protein-1 (Blimp1). J Biol Chem.

[47] Oh E, Lee HY, Kim HJ, Park YJ, Seo JK, Park JS (2015). Serum amyloid A inhibits RANKL-induced osteoclast formation. Exp Mol Med.

[48] Kassem A, Lindholm C, Lerner UH (2016). Toll-Like Receptor 2 Stimulation of osteoblasts mediates Staphylococcus aureus induced bone resorption and osteoclastogenesis THROUGH enhanced rankl. PLoS ONE.

[49] Petronglo JR, Putnam NE, Ford CA, Cruz-Victorio V, Curry JM, Butrico CE (2022). Context-dependent roles for toll-like receptors 2 and 9 in the pathogenesis of Staphylococcus aureus osteomyelitis. Infect Immun.

[50] Takayanagi H, Kim S, Matsuo K, Suzuki H, Suzuki T, Sato K (2002). RANKL maintains bone homeostasis through c-Fos-dependent induction of interferon-beta. Nature.

[51] Lee Y, Huang H, Kim HJ, Park CK, Kim HH (2008). The phosphatidylinositol 3-kinase-mediated production of interferon-beta is critical for the lipopolysaccharide inhibition of osteoclastogenesis. Life Sci.

[52] Hayashi T, Kaneda T, Toyama Y, Kumegawa M, Hakeda Y (2002). Regulation of receptor activator of NF-kappa B ligand-induced osteoclastogenesis by endogenous interferon-beta (INF-beta ) and suppressors of cytokine signaling (SOCS). The possible counteracting role of SOCSs- in IFN-beta-inhibited osteoclast formation. J Biol Chem.

[53] Lee Y, Hyung SW, Jung HJ, Kim HJ, Staerk J, Constantinescu SN (2008). The ubiquitin-mediated degradation of Jak1 modulates osteoclastogenesis by limiting interferon-beta-induced inhibitory signaling. Blood.

[54] Miron RJ, Bosshardt DD (2018). Multinucleated giant cells: good guys or bad guys?. Tissue Eng Part B Rev.

[55] Ratter JM, Rooijackers HMM, Hooiveld GJ, Hijmans AGM, de Galan BE, Tack CJ (2018). In vitro and in vivo effects of lactate on metabolism and cytokine production of human primary PBMCs and monocytes. Front Immunol.

[56] Schenz J, Heilig L, Lohse T, Tichy L, Bomans K, Buttner M (2021). Extracellular lactate acts as a metabolic checkpoint and shapes monocyte function time dependently. Front Immunol.

[57] Li B, Lee WC, Song C, Ye L, Abel ED, Long F (2020). Both aerobic glycolysis and mitochondrial respiration are required for osteoclast differentiation. FASEB J.

[58] Indo Y, Takeshita S, Ishii KA, Hoshii T, Aburatani H, Hirao A (2013). Metabolic regulation of osteoclast differentiation and function. J Bone Miner Res.

[59] Chakraborty S, Handrick B, Yu D, Bode KA, Hafner A, Schenz J (2022). Galpha(q) modulates the energy metabolism of osteoclasts. Front Cell Infect Microbiol.

[60] Aki T, Funakoshi T, Noritake K, Unuma K, Uemura K (2020). Extracellular glucose is crucially involved in the fate decision of LPS-stimulated RAW264.7 murine macrophage cells. Sci Rep.

[61] Rumbaugh KP, Sauer K (2020). Biofilm dispersion. Nat Rev Microbiol.

