# Supplementary material for: Staphylococci planktonic and biofilm environments differentially affect osteoclast formation
Source: Inflamm Res. 2023 Jun 17;72(7):1465–84. doi: 10.1007/s00011-023-01745-9 (PMC10352167; doi:10.1007/s00011-023-01745-9)
Supplement: Supplementary file 1 — Supplementary file1 (DOCX 697 KB) [file 11_2023_1745_MOESM1_ESM.docx]

***Supplementary material***

***Manuscript: Staphylococci planktonic and biofilm environments differentially affect osteoclast formation.***

***Seebach E et al. 2023***

**Suppl. Table 1 Experimental parameters.**

| **Approach** | **Stimulation time** | **Plate format** | **Cell number** | **Total volume** | **Further additives** | **Media exchange** |
| --- | --- | --- | --- | --- | --- | --- |
| **TRAP staining** | 5 - 6 days | 24 well | 5*10^3^ | 1 ml | β-ME | day 2-3/5 |
| **RT-qPCR** | 1 day | 6 well | 1.5*10^6^ | 2 ml |  |  |
| **RT-qPCR/CBA** | 2 days | 6 well | 1*10^6^ | 2 ml |  |  |
| **RT-qPCR** |  | 24 well | 1*10^5^ | 1 ml |  |  |
| **WB** | 2 days | 6 well | 1*10^6^ | 2 ml |  |  |
| **Mito copy No. /** | 4 days | 24 well | 1*10^5^ | 1 ml | β-ME | day 2-3 |
| **Mito activity** |  |  |  |  |  |  |
| **NO assay** | 2 days | 96 well | 2.5*10^4^ | 0.2 ml |  |  |
| **TLR blocking** | 1 + 24 hours | 48 well | 1.5*10^5^ | 0.5 ml |  |  |

Experiments were performed in suitable well plate formats depending on the material quantity needed for further analysis. Cell numbers were adjusted according to the respective well area and incubation time. 50 µM β-mercaptoethanol (β-ME) was added in experiments with incubation times longer than 2 days to reduce cell proliferation and avoid overgrowing. In these experiments, half of the medium was exchanged and cells were re-stimulated with half of the stimuli late on day 2 or early on day 3. In case that 6 days for differentiation were necessary, half of the medium and stimuli were additionally refreshed on day 5. Blocking and neutralizing antibodies were added freshly every 24 hours. TRAP: Tartrate-resistant acid phosphatase, RT-qPCR: reverse transcriptase quantitative polymerase chain reaction, CBA: Cytometric Bead Array, Mito: Mitochondrial, NO: Nitric oxide.


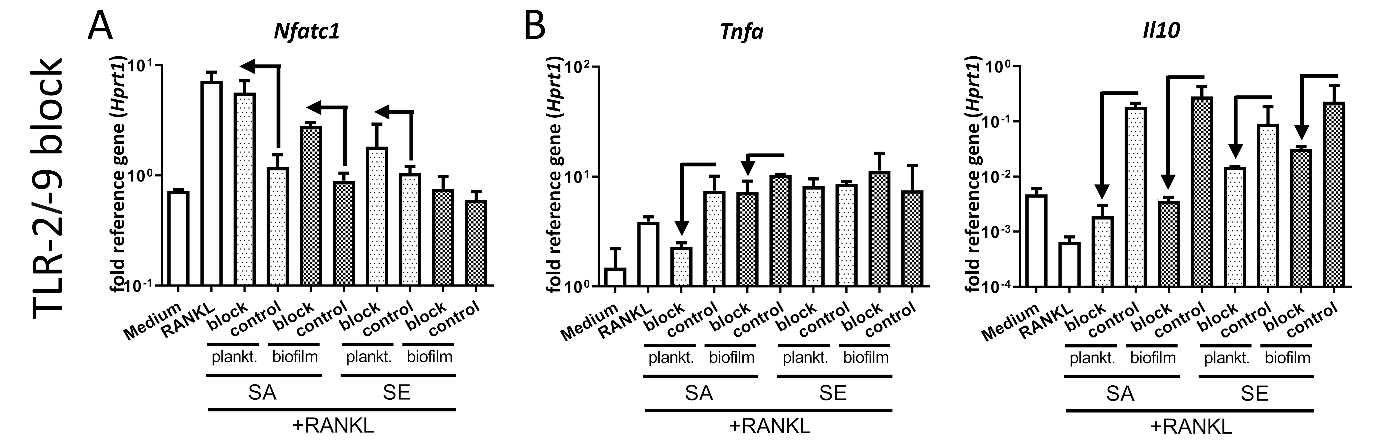


**Suppl. Fig. 1 Effect of combined inhibition of TLR-2 and TLR-9 signaling on macrophage response towards CM.** RAW 264.7 cells were pre-incubated with TLR-2 specific blocking antibody (10 µg/ml) and TLR-9 inhibitory ODN 2088 (2 µM) or respective IgG isotype and respective control ODN (all InvivoGen, USA) for 1 hour in fresh growth media (DMEM high glucose + 10% FCS + 1% Pen/Strep). Cells were then stimulated by adding CM (same volume as fresh growth media; 1:1) + RANKL (50 ng/ml) and further blocking agents for 24 hours. After stimulation time, RNA was extracted by the ExtractMe total RNA Micro Spin Kit (BLIRT S.A., Gdańsk – Poland), 1 µg total RNA was transcribed into cDNA by the enzymatics M-MuLV RT (Qiagen, MA – USA) and analyzed for gene expression of osteoclastogenesis marker NFATc1, pro-inflammatory cytokine TNF-α and anti-inflammatory cytokine IL-10 by qPCR. A+B) Relative mRNA levels of *Nfatc1* (A), *Tnfa* or *Il10* (B) after combined TLR-2 and TLR-9 blocking and stimulation by CM + RANKL for 24 hours. n=2 experiment, mean + SD are shown. Arrows indicate the effect of TLR blocking on respective CM + RANKL treatment.


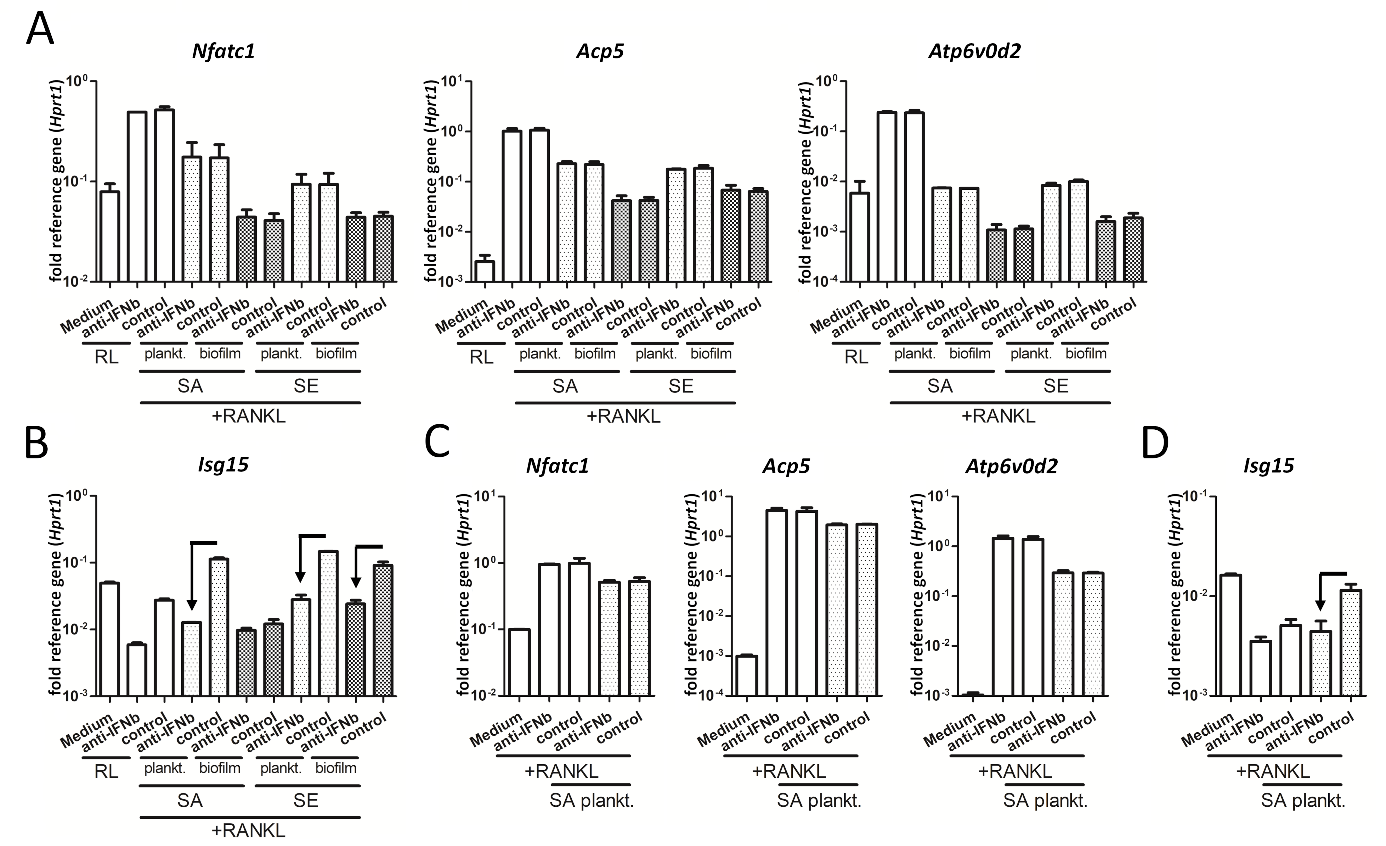


**Suppl. Figure 2 Effect of IFN-β neutralization on osteoclastogenic differentiation of macrophages.** RAW 264.7 cells were cultivated in growth media (DMEM high glucose + 10% FCS + 1% Pen/Strep) ± CM and effect of IFN-β neutralization on RANKL-mediated osteoclastogenesis of macrophages was analyzed. A+B) Expression analysis of osteoclastogenic marker genes (A) and IFN-β target gene *Isg15* (B) of un-committed macrophages. Cells were stimulated with CM and RANKL (50 ng/ml) and IFN-β neutralizing antibody (50 U/ml) or IgG control for 2 days and mRNA levels of *Nfatc1*, *Acp5*, *Atp6v0d2* or *Isg15* were quantified by RT-qPCR. Data are presented as relative gene expression of gene of interest related to the reference gene *Hprt1*. n=2 experiments. mean + SD are shown. Arrows indicate the effect of IFN-β neutralization on respective CM + RANKL treatment. C+D) Expression analysis of osteoclastogenic marker genes (C) and IFN-β target gene *Isg15* (D) of RANKL-primed osteoclast progenitor cells. Cells were stimulated with RANKL (50 ng/ml) for 2 days. On day 2, SA plankt. CM + RANKL and IFN-β neutralizing antibody (50 U/ml) or IgG control were added. On day 4, mRNA levels of *Nfatc1*, *Acp5*, *Atp6v0d2* or *Isg15* were quantified by RT-qPCR. Data are presented as relative gene expression of gene of interest related to the reference gene *Hprt1*. n=2 experiments. mean + SD are shown. Arrows indicate the effect of IFN-β neutralization on respective CM + RANKL treatment.


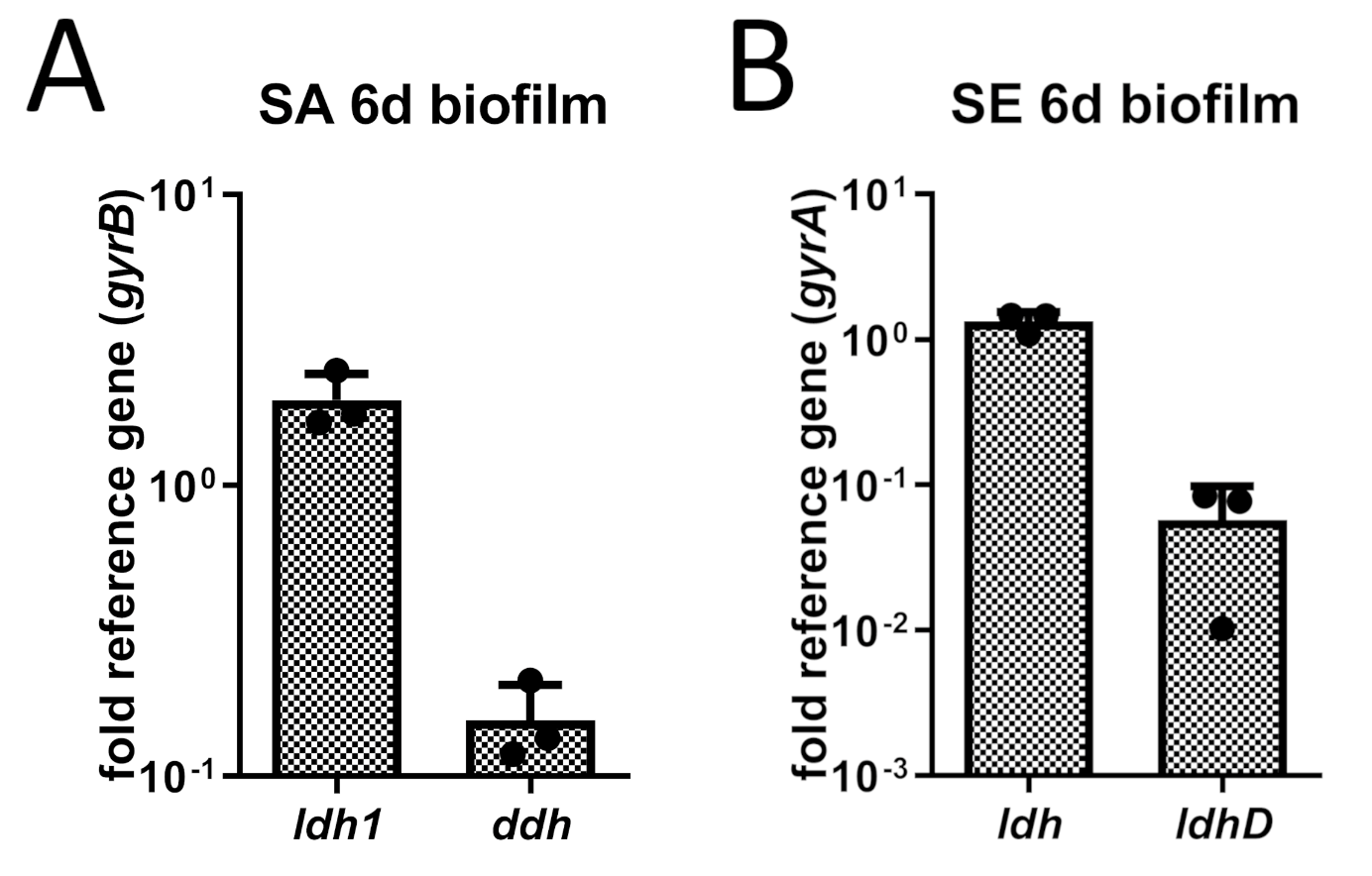


**Suppl. Figure 3 Expression of L- or D-lactate dehydrogenases in SA and SE biofilms.** SA and SE were cultured as biofilms for 6 days in cell culture medium (DMEM high glucose + 10% FCS). Biofilms were harvested by scraping and centrifugation. Biofilm matrix was destroyed by sonication in an ultrasonic bath and following glass bead disruption (MINI-BEADBEATER™, Bio Spec Products Inc.). Lysis was performed with the innuPREP Bacteria Lysis Booster (Analytik Jena, Germany) and subsequent RNA isolation according to the manufacturer’s protocol. An additional DNAse I step was carried out in the RNA samples to eliminate potential DNA contamination. RNA was transcribed into cDNA by using hexamer primers following the manufacturer’s instructions. mRNA levels of *ldh1* (SAUSA300_0235; fw: AACAACGTCCTGAGGGCAAA; rev: TGATGACTGCTGGAACACCA) and *ddh* (SAUSA300_2463; fw: AATTAGTGCGTCGCTTCCCA; rev: TGTAGCAGCACCGATACGAC) for SA and *ldh* (SERP2156; fw: GTGGCTTTGATGGTTTCTTCCT; rev: TGCAAGTTCAGAGTCACCATGT) and *ldhD* (SERP2087; fw: TACAGGGCGTATTGGTGCTG; rev: CTCCTCTTGCGGCATTGACT) for SE were quantified by RT-qPCR. Data are presented as relative gene expression of gene of interest related to the reference gene *gyrB* (SAUSA300_0005; fw: AGTAACGGATAACGGACGTGGTA; rev: CCAACACCATGTAAACCACCAGAT) for SA and *gyrA* (SERP2548; fw: AGCAGCAGGTGTGAAAGGTA; rev: TACGCTCGGTAATTGTCGCA) for SE. n=3 experiments. Data are presented as mean + SD and single values are shown as dots.
